# Therapeutic ultrasound: an innovative approach for targeting neurological disorders affecting the basal ganglia

**DOI:** 10.3389/fnana.2024.1469250

**Published:** 2024-10-02

**Authors:** Anurag Singh, John N. J. Reynolds

**Affiliations:** Translational Brain Plasticity Laboratory, Department of Anatomy, School of Biomedical Sciences, and the Brain Health Research Center, University of Otago, Dunedin, New Zealand

**Keywords:** basal ganglia, ultrasound, focused ultrasound, blood–brain barrier, therapeutics, Parkinson’s disease, neurological disorders

## Abstract

The basal ganglia are involved in motor control and action selection, and their impairment manifests in movement disorders such as Parkinson’s disease (PD) and dystonia, among others. The complex neuronal circuitry of the basal ganglia is located deep inside the brain and presents significant treatment challenges. Conventional treatment strategies, such as invasive surgeries and medications, may have limited effectiveness and may result in considerable side effects. Non-invasive ultrasound (US) treatment approaches are becoming increasingly recognized for their therapeutic potential for reversibly permeabilizing the blood–brain barrier (BBB), targeting therapeutic delivery deep into the brain, and neuromodulation. Studies conducted on animals and early clinical trials using ultrasound as a therapeutic modality have demonstrated promising outcomes for controlling symptom severity while preserving neural tissue. These results could improve the quality of life for patients living with basal ganglia impairments. This review article explores the therapeutic frontiers of ultrasound technology, describing the brain mechanisms that are triggered and engaged by ultrasound. We demonstrate that this cutting-edge method could transform the way neurological disorders associated with the basal ganglia are managed, opening the door to less invasive and more effective treatments.

## 1 Introduction

The term “basal ganglia” refers to a cluster of nuclei embedded deep within the cerebral hemispheres and includes the striatum, consisting of the caudate and lenticular nuclei (the putamen, globus pallidus externus, and internus) and nucleus accumbens, and related nuclei such as the substantia nigra, subthalamic nucleus, and ventral pallidum. These basal ganglia structures are closely associated with one another through complex synaptic connections, and, through a number of partially segregated parallel loops with the cortex, are responsible for motor control, reinforcement learning, action selection, regulation of emotions, and the performance of executive functions (Lanciego et al., 2012; Alexander et al., 1986; Redgrave et al., 2011).

The basal ganglia circuitry is composed of input and output nuclei with extensive intrinsic processing. The striatum is the major input area of the basal ganglia and is divided into two main structures, the dorsal striatum and the ventral striatum. The striatum receives glutamatergic input from the cortex, the intralaminar thalamic nuclei, and dopaminergic input from the substantia nigra, and the ventral tegmental area. The output nuclei of the dorsal striatum consist of GPi and SNpr, which transmit inhibitory projections to the thalamus. Specifically, the dorsal striatum output through GABAergic projections of spiny projection neurons to the GPe is known as the “Indirect pathway,” or to the GPi as the “Direct pathway.” The ventral striatum consists of the nucleus accumbens and olfactory tubercle, which project to the ventral pallidum and SNpr and receive inputs from limbic areas, thalamus and other areas (Lanciego et al., 2012).

The basal ganglia receive information from the cortex, processes it, and determines which activities to “release” or “disinhibit” (Mathai and Smith, 2011). Dysfunction of the basal ganglia is often manifested in movement disorders such as Parkinsonian syndrome consisting of rigidity, akinesia/bradykinesia and resting tremor, dystonia due to sustained muscle contraction and abnormal posture, and chorea-ballism where brief movement fragments appear to flow irregularly from one body segment to the other in a dance-like appearance (Magrinelli et al., 2016).

In the last two to three decades, there has been a tremendous growth in knowledge of the pathophysiology of movement disorders including the progressive dopamine deficiency manifested as Parkinson’s disease (PD), its symptomatic management, and the effectiveness and rationale for emerging treatments. Treatments for PD and other basal ganglia disorders that have found their way into clinical application include medication, physiotherapy, surgical interventions such as deep brain stimulation, cell replacement therapies (to restore dopamine in PD), and gene therapy approaches, among many others. However, researchers have yet to develop effective preventive or curative therapies.

One major obstacle to the availability of more effective pharmacological treatments for basal ganglia disorders is the presence of the blood–brain barrier (BBB), a semi-permeable membrane serving as a neuroprotective structure, ensuring homeostasis inside the brain. The cerebrovascular system of the BBB is tightly interlinked and prevents the entry of large drugs or molecules (> 400 Daltons) inside the brain (Pardridge, 2012). Therefore, it is difficult for large drugs lacking a specific transport mechanism to cross the BBB and enter the brain parenchyma. Another challenge is the anatomical localization of the basal ganglia, which is deeply embedded inside the brain. Contrary to superficial brain layers, the basal ganglia nuclei are positioned below the cerebral cortex, nestled within the white matter, and surrounded by the thalamus and internal capsule. Due to the deep positioning of the basal ganglia and the relatively small size of the nuclei, it is challenging to access by surgical or functional imaging/EEG approaches, and hence, difficult for researchers and clinicians to effectively study and treat this area.

Recent advances in treatment approaches have ushered in a new age in therapy, with methods such as transcranial ultrasound (US) becoming powerful alternatives for modifying basal ganglia function. In the 1950s, ultrasound treatment was first applied in the context of psychiatric disorders by a visionary neurosurgeon named Lars Leksell (Leksell, 1956). The application of ultrasound to movement, psychiatric disorders, and brain tumors was then led by Meyers et al. (1959) and Nelson et al. (1959). Technological developments in transducer technology and simultaneous imaging have led to an increase in research using ultrasound over the last two to three decades. The transcranial ultrasound has opened up a wide vista of possibilities for application to neurodegenerative, neuropsychological, and neoplastic disorders. This review examines the research behind this innovative application of ultrasound and how it could be used to treat brain disorders related to the basal ganglia. This review will discuss therapeutic ultrasound, the BBB, drug delivery using ultrasound as a stimulus, and its significance in diseased conditions.

## 2 Emerging frontiers in the treatment of basal ganglia disorders and other neurodegenerative conditions with focused ultrasound

Basal ganglia disorders, such as PD, pose significant difficulties for patients and their families, leading to a substantial social and economic burden. Despite many decades of research, its prevention or long-term effective treatment remains a challenge. There exists an incomplete understanding of the etiology of PD and its pathogenesis leading to (i) a short therapeutic window for effective drug treatment in many cases, (ii) an inability to accurately identify the disorder at preclinical stages due to a lack of a minimally invasive and widely accessible biomarker, and (iii) a failure to deliver disease-modifying drugs to the affected and/or degenerating brain in a targeted and physiological manner.

The characteristic primary pathology of PD is the progressive degeneration of dopaminergic neurons in the substantia nigra, resulting in a steady decrease in dopamine delivery to the striatum and other frontal areas (Dauer and Przedborski, 2003). Motor symptoms of PD can be well managed in the initial years with dopaminergic drugs, particularly levodopa (Lewitt, 2015). In the later stages, when the side effects associated with drug therapy are no longer manageable, symptom improvement can be achieved through deep brain stimulation (DBS) and focal lesioning by radiofrequency, gamma knife, and other methods (Higuchi et al., 2017; Schreglmann et al., 2018). All currently used therapeutic approaches beyond standard drug treatment continue to be primarily symptomatic and invasive, and no treatment can currently halt or at least mitigate the process responsible for the pathogenesis of PD.

In PD, the brain nuclei that hold promise for targeting with focused ultrasound (FUS) are the subthalamic nucleus (STN), globus pallidus interna (GPi), and the ventral intermediate nucleus (Vim) of the thalamus. When a patient is medically unable to undergo an open surgical procedure, for example, in elderly patients with significant medical co-morbidity, the recommended course of action may now be ablating part of the basal ganglia circuit using FUS, with targeting guided by magnetic resonance imaging (MRgFUS). MRI coupled with FUS has already crossed the transition phase from the laboratory to the clinic. Specifically, FUS thalamotomy is both effective and FDA-approved for the treatment of Parkinsonian tremor (Elias et al., 2013; Bond et al., 2017); FUS pallidotomy (ablation of GPi) has yielded preliminary efficacy in treating dyskinesias and motor fluctuations in advanced PD (Jung et al., 2018); and according to a pilot study done in 2018, FUS subthalamotomy demonstrated positive clinical effects on PD-related abnormalities (Martínez-Fernández et al., 2018).

In addition to PD, FUS has found significant application in alleviating tremors without any underlying neurodegenerative disorder, so-called essential tremor (ET). MRgFUS-thalamotomy resulted in a tremor score reduction of 89.4 and 81% (at 1 and 3 months, respectively) post-treatment in four patients with refractory ET (Lipsman et al., 2013). Another study reported a 74.5% reduction in the hand tremor scale in 15 ET patients who were followed up for a year post-treatment (Elias et al., 2013). According to a multicentre, randomized, controlled clinical trial involving 76 ET patients, the participants were allocated to either unilateral MRgFUS-thalamotomy or sham surgery in a 3:1 ratio. In the 3-month assessment, hand tremor scores were improved by 47%, while the sham procedure scores had minimal improvement of only 1%. However, the benefit slightly declined by the 12-month follow-up, when the improvement was noted to be 40% better off than the baseline (Elias et al., 2016). The advanced FUS-based non-invasive treatment of deep brain tissue has found lasting clinical application for the motor disorders associated with ET and PD.

Similarly, in Alzheimer’s disease (AD) and other dementias, FUS has emerged in the last decade as a distinctly promising non-invasive therapeutic intervention. Nicodemus et al. (2019) published an important study investigating transcranial FUS stimulation in dementia patients. A 2-MHz transducer was used to administer FUS treatment for a duration of 1-h to the medial temporal lobe, under the guidance of MRI and Doppler ultrasound. The patients had no severe side effects, and 63% of them showed significant positive changes in cognitive assessments such as the Clinical Dementia Rating (CDR) and the Montreal Cognitive Assessment (MoCA). Furthermore, 9.1% of the patients showed positive changes in gross motor functioning after 8 weeks.

In a clinical trial (Beisteiner et al., 2020), subjects with probable AD were treated with ultrashort ultrasound pulse stimulation, with 6,000 pulses of 3 μs duration administered at 5 Hz in each session. The treatment consisted of three sessions within 2–4 weeks and involved stimulating the dorsolateral prefrontal cortex and associated areas. In a 3-month follow-up MRI, the patients experienced no clinical side effects such as tissue damage, hemorrhage, oedema, or changes in intracranial pathology. Based on the results of clinical studies, the cognitive function of the patients was significantly improved and was found to be stable for at least 3 months post-treatment. Functional MRI (fMRI) confirm an increase in memory network and hippocampus activity, which is associated with cognitive benefits in patients with AD. It will be interesting to see if this application of FUS can be used for the long-term treatment of other dementias, such as those associated with PD. Other than PD and AD, FUS has been found effective in the treatment of obsessive-compulsive disorder (OCD; Jung et al., 2015), major depressive disorder (Kim et al., 2018), chronic pain (Kim et al., 2018), and brain cancers (Coluccia et al., 2014).

## 3 Therapeutic ultrasound

Ultrasound, when used for the treatment of diseased organs or body tissues, is referred to as therapeutic ultrasound. When used as a non-invasive medical tool, therapeutic ultrasound provides clinicians with a powerful, easily accessible, and relatively cheap diagnostic imaging technique. Therapeutic ultrasound has become a non-invasive modality for treating brain disorders, impacting neural function without the need for surgical intervention. Utilizing the effects of ultrasound on tissues and drug delivery vehicles, the aim is for the user to be able to alter neuronal activity within targeted brain regions, offering therapeutic potential across a diverse range of neurological and neuropsychiatric disorders. Here we focus on the effect of ultrasound at neurotransmitter and cellular/molecular levels and its potential application to PD and other disorders.

Ultrasound can be used to adjust brain function by stimulating or inhibiting specific brain regions to modulate neuronal activity. This can be achieved using FUS, wherein ultrasound waves are directed through the intact skull to target specific brain regions. By carefully designing the transducers and parameters, ultrasound energy can be focused to directly stimulate neurons in targeted brain regions, such as the deep brain nuclei of the basal ganglia. This alteration in brain function provides the ability to non-invasively modulate neuronal activity and achieve lasting alteration of neural networks.

### 3.1 Principles of therapeutic ultrasound

**Figure 1:**
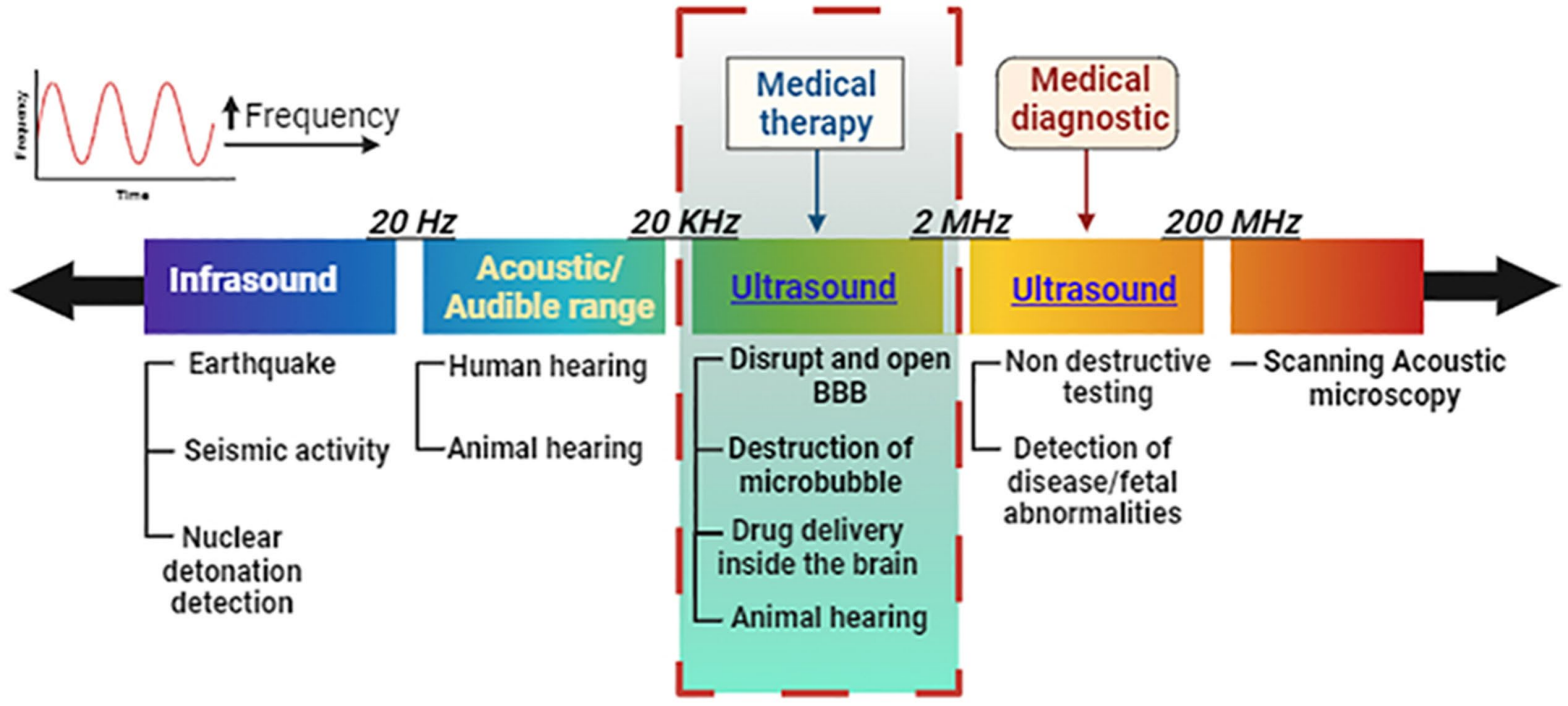
Frequency discrimination in the ultrasound spectrum for audible range and medical therapy vs. diagnostic conditions. Ultrasound for medical treatment can open the blood–brain barrier and deliver or release drugs under controlled conditions at specific regions inside the brain. Image created in Biorender.com.

Ultrasound is defined as sound waves (mechanical oscillating pressure waves) with frequencies greater than the 20 kHz threshold for human hearing (Figure 1). Ultrasound may be administered to the human body at various frequencies and was first used in medicine to cure diseases before being used to detect them (Haar and Coussios, 2007). Each spectrum affects bodily tissues differently and penetrates to varying depths (Mason, 2011; Gourevich et al., 2013).

Focused ultrasound is generated using piezoelectric transducers capable of inducing acoustic pressure under alternating current stimulation. Piezoelectric materials include quartz, polymers, and ceramics (Gibbs et al., 2009). As the miniaturization of piezo-active elements progressed (e.g., with the application of lead zircon), transducers became appropriate for use in medical applications. For FUS, curved spherical transducers are used, in which the focal distance is determined by the radius of curvature and the diameter of the transducer (Malietzis et al., 2013). This can, however, be altered in a bespoke design to customize the transducers for use in rats, mice, sheep, non-human primates (NHPs), and humans (Deffieux et al., 2013; Guo et al., 2023; Chen et al., 2024; Pelekanos et al., 2018).

Transcranial FUS was made feasible during the early 2000s by combining ultrasound transducers with real-time MRI thermometry (Hynynen et al., 2006). However, due to the nonuniform thickness and the inhomogeneous composition of the skull bones, phase aberration of the ultrasound beam can occur, which can distort the therapeutic focus and reduce the efficiency of the treatment (Chang et al., 2016). High ultrasound absorption results in undesirable heating of the skull, which can also lead to thermal injury of the underlying cerebral cortex. Since then, phased array transducers employing multiple elements have been designed to correct and overcome targeting discrepancies due to skull structure and variability during US delivery, guided by advanced bone imaging using CT (Aubry and Tanter, 2016).

## 4 Clinical application of therapeutic ultrasound

Through the therapeutic use of ultrasound, which is under intensive investigation in humans, some of the issues related to the treatment of disorders such as PD and other basal ganglia disorders could potentially be addressed. We shall now discuss the physiological effects of therapeutic ultrasound, which are being harnessed for clinical applications.

### 4.1 Ablation

As introduced in an earlier section, currently the most clinically utilized of all the bioeffects of FUS is thermal ablation, which annihilates cells in the targeted area with minimal effect on neighboring cells and tissues. With ultrasound ablation, high-intensity ultrasound signals radiate from many hundreds of transducer elements and converge on a desired volume of tissue within the intracranial region. The degree of thermal ablation achieved is dependent on the time and temperature during FUS treatment (Sapareto and Dewey, 1984; Vigen et al., 2006). Mechanical energy from the ultrasonic waves is focused on the region of interest and is transformed into heat, thereby causing protein coagulation and cell death when the cumulative thermal index is adequate (O’brien, 2007, Haar and Coussios, 2007). Temperatures above 56°C are primarily used to achieve tissue necrosis (Mouratidis et al., 2019). Between 55 and 60°C, the transducers can target tissues as small as cubic millimeters (nominally 1 mm^3^ at minimum), causing coagulation and denaturation of protein in a matter of seconds (Sapareto and Dewey, 1984; Zhou, 2011).

### 4.2 Cavitation/blood–brain barrier permeabilization

**Figure 2:**
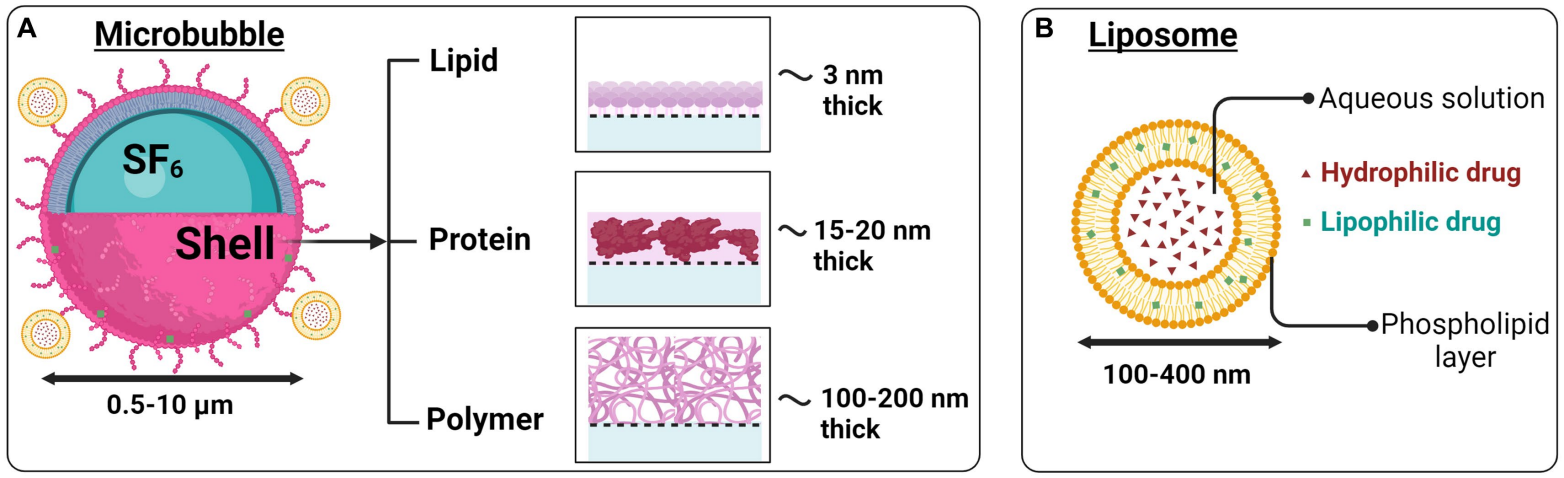
Microbubbles and liposomes as ultrasound-based drug delivery systems. **(A)** Therapeutic agents can be attached to gas-filled microbubbles or **(B)** liposomes having a hydrophilic core and lipophilic outer layer. Image created in Biorender.com.

The BBB is a physiological structure that isolates the brain’s extracellular environment from the circulatory system perfusing the rest of the body. It consists of perivascular astrocyte end feet, endothelial cells, and a basement membrane. As the name suggests, the BBB prevents the movement of the majority of molecules inside the brain, thus maintaining brain homeostasis. It regulates the influx and efflux transport of ions/molecules and protects neurons and glial cells inside the brain (Zhao et al., 2015; Obermeier et al., 2013; Banks, 2016; Wu et al., 2023). Focused ultrasound offers the therapeutic possibility of accurately and momentarily opening the BBB in a specific location. It is non-invasive and can be combined with other therapeutic approaches, such as microbubbles or liposomes, as shown in Figures 2A,B, to diagnose or treat certain disease conditions (Burgess et al., 2015; Barzegar-Fallah et al., 2022; Gandhi et al., 2022).

Because ultrasound waves lead to a transfer of momentum, they can themselves generate acoustic radiation force, microstreaming, and shear stress to produce mechanical effects (Nowicki, 2020; Miller et al., 2012). However, the mechanical effects of ultrasound are increased significantly when the ultrasonic waves hit microbubbles. This decreases the acoustic energy required to be delivered for BBB opening, thereby improving the overall safety profile of FUS for this application (Dayton et al., 1999; Kim et al., 2023). Microbubbles are FDA-approved ultrasound contrast agents with a diameter of 1–10 μm (Burgess et al., 2011; Kobus et al., 2016). They primarily consist of a gaseous sulfur hexafluoride (SF_6_) or perfluorocarbon (PFC) core (Figure 2A), surrounded by proteins or lipid shells (Sirsi and Borden, 2009). When FUS is accompanied by the real-time intravenous injection of microbubbles, the microbubbles oscillate in phase with the ultrasound waves, accentuating microstreaming events and temporarily causing disruption and opening of the adjacent BBB (Parodi et al., 2019). Thermal effects are also likely present but are not thought to be the primary mechanism of BBB opening when FUS and microbubbles are combined (Hynynen et al., 2001). For a more detailed discussion of this topic, the reader is referred to other specialist reviews (Barzegar-Fallah et al., 2022).

Preclinical and clinical studies utilizing FUS with microbubbles have demonstrated that FUS-driven BBB opening for drug delivery is safe and efficient if the acoustic irradiation parameters and microbubble concentrations are closely supervised (McMahon and Hynynen, 2017). However, a notable drawback of microbubbles is that they have low retention of hydrophobic drugs and extremely short circulation times *in vivo* (Xie et al., 2009).

Reversibly permeabilizing the BBB using microbubbles is under active investigation as a means to deliver large molecules into the brain parenchyma. Most commonly, the aim is the delivery of large chemotherapeutics to treat brain cancers. The FUS technology has been approved by the FDA for some types of cancer therapy, namely, brain cancer, prostate cancer, uterine leiomyoma, and bone metastases, and it is being researched for other neoplasms (Bachu et al., 2021). There have been many studies of BBB opening with FUS in glioblastoma models in both small and larger animals that demonstrate a high degree of safety as well as effectiveness. In animal studies, acute flow-through of the BBB is reversible within 6–8 h, without any evidence of axonal or neuronal injury. According to a study published in 2001, in conjunction with intravenous microbubbles, FUS (< 1.5–2 MPa) induces transient, repeatable, and localized BBB opening in rabbits without any noticeable neuronal damage (Hynynen et al., 2001). In animal models, FUS has been effective in treating brain cancer by improving the brain transport of several cancer therapy medications, including doxorubicin (Aryal et al., 2015), carboplatin (Dréan et al., 2019), methotrexate (Mei et al., 2009), and trastuzumab (Dréan et al., 2019). For example, FUS enhances doxorubicin diffusion across the BBB and facilitates advanced tumor control. It also improves animal survival (Sun et al., 2017; Kovacs et al., 2014). FUS-generated delivery of doxorubicin in liposomes (Figure 2B) can lower side effects and prolong circulation. Notably, FUS can enhance the preloaded encapsulation of doxorubicin in liposomes (aka Doxil) through the BBB (Treat et al., 2007), and has also been shown to increase rat glioma survival (Treat et al., 2012).

Despite abundant preclinical evidence, the efficacy of FUS-mediated BBB disruption for enhanced drug delivery in humans remains limited. The first-in-human BBB opening was achieved by the surgical placement of a transducer into the epidural space directly overlying the tumor in patients with recurrent glioblastoma. The patients were treated monthly with FUS and an IV injection of microbubbles for BBB-opening, and its disruption was noted to occur at >0.8 MPa FUS pressure amplitudes (Carpentier et al., 2016).

In 2019, researchers carried out the first human trial on noninvasive MRgFUS BBB opening in malignant glioma patients with concomitant systemic administration of temozolomide chemotherapy. The patients were subjected to craniotomy and tumor resection approximately 24 h after FUS and chemotherapeutic agent treatment (Mainprize et al., 2019). Histopathological histology confirmed an increase in the signal intensity of 15–50%, reflecting transient opening of the BBB in the target tissue.

A novel device that integrates neuronavigation with FUS, the Neuronavigation-guided focused ultrasound (NaviFUS), when combined with systemic microbubbles, potentially allows delivery of the ultrasound energy accurately and in a temporally controlled manner at specific locations within the central nervous system (CNS). Although still in the early stages of clinical testing, this device can potentially enhance drug delivery for tumor control such as glioblastoma and other disease conditions with minimal side effects (Chen et al., 2020a).

This principle of FUS-induced opening of the BBB is being used as a potential disease-modifying strategy with gene therapy (Blesa et al., 2023). Gene therapy exploits various strategies, including the repair of pathogenic pathways, neuroprotection, and neurorestoration. Therefore, it holds the potential to be effective in neurodegenerative conditions such as PD, AD, and others. This is often employed using an adeno-associated virus (AAV) vehicle for gene transfer (Bedbrook et al., 2018). Recently, intravenous AAV delivery following BBB opening with FUS targeting was explored in brain regions associated with PD in normal and Parkinsonian NHPs. This study reported that the application of BBB opening with FUS in areas of PD in NHPs and human patients was safe and could be achieved with high accuracy toward predetermined targets (Blesa et al., 2023). They also concluded that systemically administered AAV vectors efficiently target focal areas in the brain of adult NHPs, thus boosting neuronal protein expression that can be used for therapeutic target validation. However, it has been difficult to achieve a higher level of transgene expression in the NHP brain compared to rodents, following repeated administration of systemic vectors (Blesa et al., 2023).

Two FDA-approved AAV-based drugs, Luxturna and Zolgensma, are under clinical trials to treat patients with genetic and non-genetic disorders (Maguire et al., 2021; Kotulska et al., 2021). Particularly, the AAV-gene therapy approach is a hot topic and is currently used in a few clinical trials involving different target regions of basal ganglia to treat PD. For example, for PD patients living with moderate to severe symptoms, clinical trials using the AAV serotype delivered by bilateral intraputamenal (Heiss et al., 2019; Muramatsu et al., 2010; Christine et al., 2022; Christine et al., 2019), and unilateral subthalamic nuclei infusion (Kaplitt et al., 2007; Niethammer et al., 2017) have either been conducted or are ongoing. Furthermore, clustered regularly interspaced short-palindromic repeat (CRISPR) based gene-editing therapeutics could directly be delivered into the brain using AAV vectors, enhancing our strategies to directly combat disease pathologies and progression (Wang et al., 2019; Li and Samulski, 2020). The disadvantage of using high doses of AAV delivery, as frequently observed with systemic AAV-gene therapy delivery, pertains to the activation of the complement or immune system, resulting in a significant inflammatory reaction (Zaiss et al., 2008). Therefore, the ability to attenuate the immune response to enhance vector delivery, for instance by using neutralizing antibodies, will facilitate an increase in the number of participants eligible for trials, and likely accelerate future research in this field.

### 4.3 Mechanical

In the 1960s, Bangham et al. discovered liposomes, which later became the most widely used nanomedicine carrier in targeted drug delivery systems (Bangham et al., 1965). Liposomes are a candidate drug carrier biomolecule because of their versatility and unique modifiable characteristics (Huang and MacDonald, 2004; Hinow et al., 2016; Mujoo et al., 2015; Regenold et al., 2022; Maruyama et al., 2022). Liposomes are nanoparticles, in the range of 10 s to 100 s nm in diameter and are typically composed of an outer shell made up of biocompatible phospholipids that can carry lipophilic drugs and an inner aqueous solution core that can carry hydrophilic drugs (Figure 2B). The lipid content and the characteristics of the shell, such as stiffness and surface charge, have a significant impact on drug discharge, surface-to-volume ratio, biodistribution, and pharmacokinetics (Cheng et al., 2021; Sharma et al., 2021).

**Figure 3:**
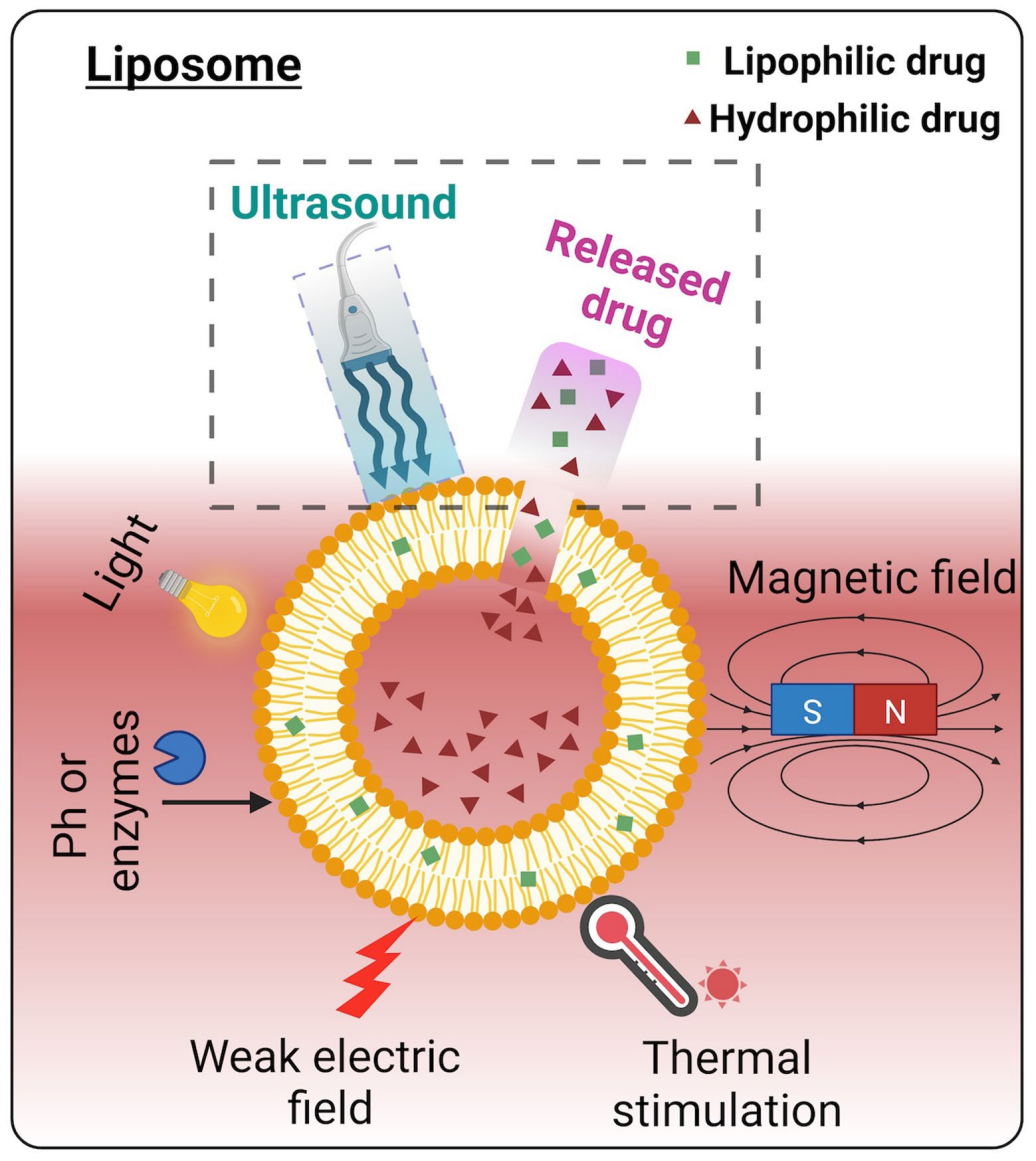
Various triggering mechanisms of drug release from the liposome. Among these energy triggers, ultrasound has been widely acknowledged as a non-invasive and facile procedure for controlled drug release. Image created in Biorender.com.

The advantages that liposomes possess compared to other nano-sized drug carriers include enhanced drug stability, enhanced bioavailability, and enhanced tissue absorption (Sercombe et al., 2015, Barenholz, 2001; see Figures 2A,B). In addition to the above, liposomes have made considerable progress in recent years in carrying peptides, proteins (Watson et al., 2012), and nucleic acids (Dos Santos Rodrigues et al., 2019). To get drug release from liposomes, stimuli such as pH, temperature, radiation, and electromagnetic energy have been subjected to intensive research as targeted therapies (Blackmore et al., 2019; Aryal et al., 2015) (Figure 3). Ultrasound energy has also been recently recognized as a relatively non-invasive and facile control stimulus for the delivery of therapeutics to the brain (Kim et al., 2022; Mackay et al., 2019; Barzegar-Fallah et al., 2022; Nakano et al., 2022).

So, how does ultrasound enhance drug delivery for circulating nanocarriers, such as liposomes? Local hyperthermia may improve drug delivery to targeted areas by exceeding the phase transition temperature of the phospholipid bilayer, thereby releasing the nanocarrier content at the site. However, temperature elevation must be mild to avoid tissue damage and be clinically acceptable. Thus, mechanical effects are a significant mechanism of drug release from these carriers. The exact mechanical mechanism is still unclear; however, it is likely that there is the formation of transient pores that subsequently rupture the liposomes (Lin and Thomas, 2003), mimicking what occurs in the cavitation of cell membranes following ultrasound sonication.

## 5 Neuromodulation: ultrasound mechanism for clinical applications

Depending on the pulse delivery protocol, the FUS technique has been demonstrated to non-invasively and directly modulate neural activity in various species, including mice (King et al., 2014), rats (Kim et al., 2014), rabbits (Yoo et al., 2011), monkeys (Deffieux et al., 2013), and humans (Legon et al., 2012). One of the very first pieces of evidence for ultrasound-induced suppression of neuronal activity dates back to 1958, when, after sonicating for more than 20 s, evoked potentials were suppressed (Fry et al., 1958). In general, shorter pulses have been shown to have suppressive effects, as evidenced by the suppression of visual activity induced by light stimulation (Yoo et al., 2011), propagation of depolarization waves in rat neuronal networks (Fry et al., 1958) and brief dilation of the pupils in cats (Ballantine et al., 1960). In addition, it has been reported that ultrasound-mediated inhibition of firing rate or evoked potentials has been observed. They include the inhibition of sensory evoked potentials in the thalamus in swine (Dallapiazza et al., 2018), decreased epileptiform electroencephalogram (EEG) bursts (Min et al., 2011), and suppressed epileptic spike in PTZ-injected epileptic rats (Chen et al., 2020b), mice (Yang et al., 2020), and macaque monkeys (Lin et al., 2020), resulting in reduced epilepsy-like behavior.

Using different sonication parameters, ultrasound-mediated stimulation of neurons has been reported both *in vitro* and *in vivo.* For example, higher firing rates of primary hippocampus neurons were observed by microelectrode array recordings after ultrasound stimulation (Tasaki, 1995; Kim et al., 2017). Furthermore, some studies report ultrasound-induced motor responses (Yuan et al., 2020), sensory responses (Kim et al., 2014), depolarization of the pyramidal cell in mice’s barrel cortex (Moore et al., 2015), and action potential induction and evoked movement behaviors (Li et al., 2019). A few studies in the visual cortex and somatosensory areas of macaque monkeys have shown that ultrasound produces a modulatory effect where different neurons respond differently to the same stimulation (Wattiez et al., 2017; Yang et al., 2021).

**Figure 4:**
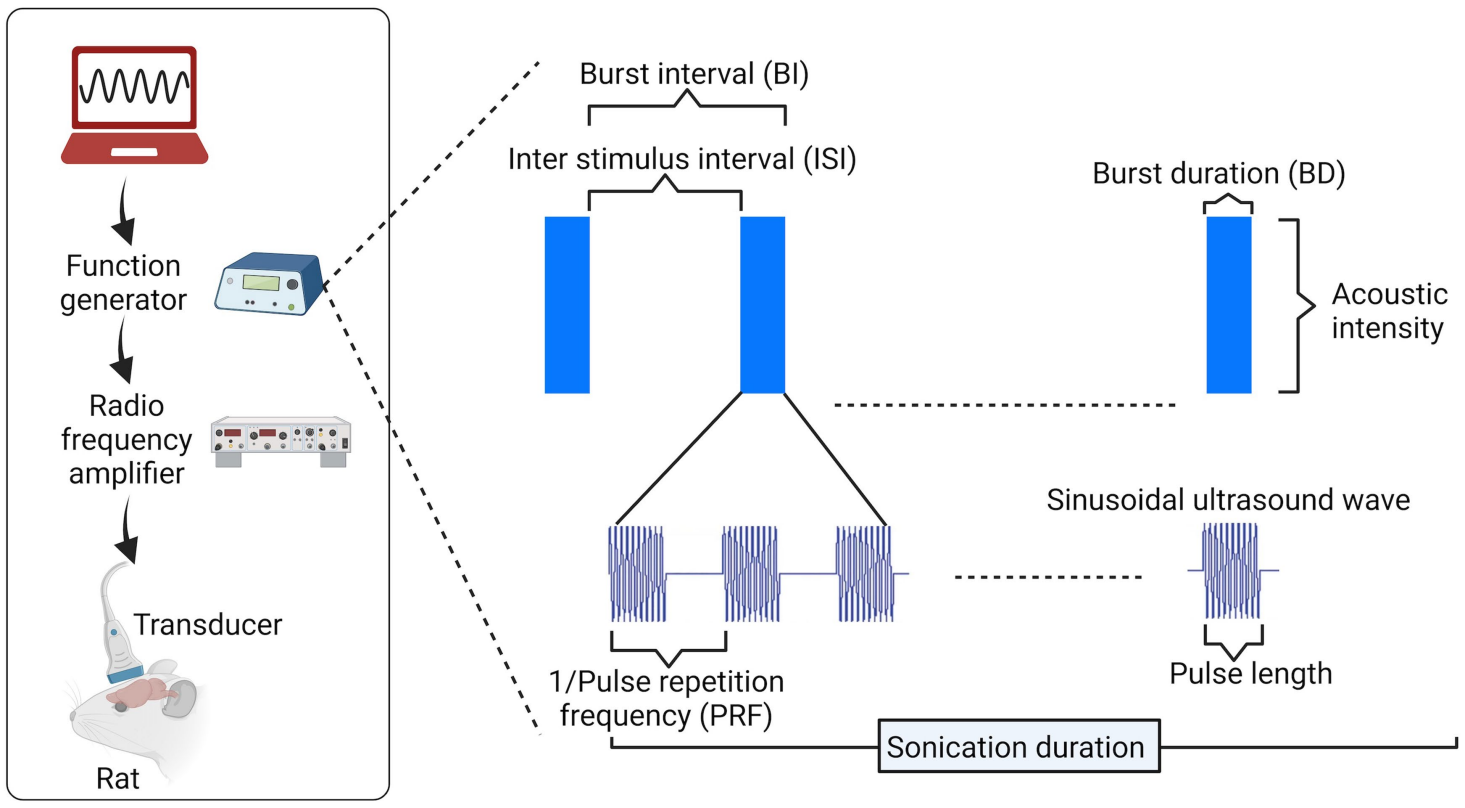
Pulse delivery parameters in ultrasound. To implement ultrasound neuromodulation (rat head as an example in this image), several parameters can be varied for pulse design, including pulse length and frequency of cycles within each pulse, pulse repetition frequency (PRF) within a burst, duty cycle [DC = burst duration (BD)/burst interval (BI)], and others. Image created in Biorender.com.

Ultrasound stimulation has also been effectively used in humans, demonstrating significant effects akin to those shown in animals. For example, following 15 s of ultrasound stimulation at 8 MHz, significant improvement in mood at 10 and 40 min was observed in chronic pain patients (Hameroff et al., 2013), restoration of consciousness with significant behavioral responsiveness post-ultrasound treatment in severely brain-injured patients (Monti et al., 2016), and improved sensory discrimination ability in unimpaired participants (Liu et al., 2021). Interestingly, ultrasound reduces pain sensitivity in healthy participants (Badran et al., 2020), and causes effective neuronal inhibition (amplitude of single-pulse motor evoked potentials), leading to advantages in motor tasks such as reduced stimulus–response reaction time (Legon et al., 2018). Figure 4 illustrates the pulse delivery parameters utilized in the FUS.

Despite the breakthroughs achieved in neuromodulation by FUS, the exact mechanisms remain unclear. Several hypotheses have been posited, both experimentally and numerically; however, the interaction at the sub-cellular, molecular, or neurotransmitter level is still unknown. In this section, we focus on some of the mechanisms proposed to explain the role of ultrasound in neuromodulation through membrane interaction, ionic change, and activation of signaling cascades.

### 5.1 Ionic/channel level

Ultrasound waves possess energy and can release heat in the medium they are traveling through. Ultrasound influences neurons (and cells in general) via the interaction with cell membranes, which are composed of lipids. Alterations in cell membrane properties such as thickness (Heimburg, 2012), curvature (Petrov, 2002) and the conformational state of lipids (Taylor et al., 2017) affect cellular capacitance, which can cause stimulation of nerve impulses (Heimburg, 2012; Luan et al., 2014).

Ultrasound has been reported to generate capacitive currents in pure lipid membranes (Prieto et al., 2013), which may be attributed to electric polarization or conformational changes (Prieto et al., 2013). Another hypothesis is that nucleation and propagation of the cavitation, which can occur in or near the lipid bilayer, may produce capacitive currents. However, the details of cavitation with the use of ultrasound in nervous stimulation remain poorly understood (Plaksin et al., 2016). Furthermore, changes in the conformation of lipids, macromolecules and channel proteins may modulate the activity of ion channels and result in neuromodulation (Perozo et al., 2002; Seeger et al., 2010).

In 2008, it was first published that ultrasound was capable of directly potentiating synaptic transmission in the CNS, as well as enhancing the influx of Ca^2+^ and Na^+^ ions (intracellular) in central neurons, leading to action potentials (Tyler et al., 2008). One proposed hypothesis is that ultrasound can stimulate neural activity by mechanically changing the state of mechanosensitive ion channels embedded in the cellular membranes at the time of transmission in neuronal tissue. These mechanosensitive proteins, due to their transmembrane location are exposed to tissue compression, stress or shear forces from ultrasound, which can generate conformational changes such as transient small cracks and mechanical deformations, enhancing the probability of the channels opening. This, in turn, will cause ion flow, depolarization, and the triggering of voltage-gated ion channels, which will result in the generation of action potentials (Tyler, 2012). However, this hypothesis has not been conclusively proven due to experimental challenges. A few groups have reported that FUS exposure may activate voltage-gated Na^+^, Ca^2+^, and K^+^ channels (Tyler et al., 2008; Kubanek et al., 2016). Despite these findings, the specific methods through which ultrasound alters the ionic content and its distribution are not well determined and thus need further examination.

### 5.2 Cellular and molecular level

Other cellular and molecular changes induced by ultrasound have been reported. Low-intensity pulsed ultrasound (LIPU) delivery to deep brain regions may activate neural networks and alter the levels of endogenous brain-derived neurotrophic factor (BDNF), with clinical application (Tufail et al., 2010). In an *in vivo* rat model of AD, Lins et al. reported that 1 MHz frequency, 1 Hz pulse repetition frequency (PRF), 50 ms burst duration (BD), and 5% duty cycle (DC) increased the level of BDNF, glial-derived neurotrophic factor (GDNF), and vascular endothelial growth factor in the lateral hippocampus after 15 min of stimulation (Lin et al., 2015). Another study reported that ultrasound (1 MHz frequency, 1 Hz PRF, 5% DC) led to the activation of NF-κB in astrocytes and promoted the expression of BDNF through the TrkB/PI3K/Akt signaling pathway and the calcium/CaMK pathway (Liu et al., 2017) *in vivo*. LIPU can also stimulate PI3K/Akt and erk-creb-trx-1 signaling pathways that can promote neurotrophic factor-induced neuronal axon growth and hence can be used for the treatment of neurodegenerative diseases (Zhao et al., 2016).

A study in 2014 reported that LIPU could promote nerve regeneration and participate in the regulation of nitric oxide synthesis, thereby promoting the differentiation of neural stem/progenitor cells into neurons. This effect improves the vitality and proliferation of iPSCs-derived neural crest stem cells and modulates the multipotential differentiation of mesenchymal stem and progenitor cell lineages through the ROCK-Cot/Tpl2-MEK–ERK signal transduction pathway (Kusuyama et al., 2014). In addition, FUS stimulated mesenchymal stem cell proliferation by enhancing the MAPK/ERK and PI3K/Akt pathways, as well as cell cycle proteins (Ling et al., 2017).

With particular relevance to basal ganglia disorders, in an animal model of PD, continuous ultrasound stimulation of the STN or GP for 30 min per day for 5 weeks yielded enhanced motor behaviors and attenuated the sustained activation of microglia and astrocytes (Zhou et al., 2021). Furthermore, FUS stimulation of the motor cortex for 7 consecutive days can also directly or indirectly improve the disability of Parkinson’s motor dysfunction through an antioxidative effect, by restoring T-SOD and GSH-PX levels (Zhou et al., 2019). Overall, these pieces of evidence indicate that US can be used in the treatment of neurodegenerative disease by interacting with glial cells, reactive oxygen species (ROS), and stem cells, and by enhancing antioxidant and neurotrophic effects, such as via increased BDNF and GDNF.

## 6 Ultrasound and synaptic plasticity

The ability of synapses to strengthen or weaken over time in response to neural activity or a stimulus is known as synaptic plasticity. The strengthening of synapses is known as long-term potentiation (LTP), while the weakening of synapses is known as long-term depression (LTD). Both LTP and LTD are widely acknowledged as physiological correlates of memory formation in the brain (Morris et al., 1986; Bliss and Gardner-Medwin, 1973).

Using western blotting, electrophysiology, and Golgi staining, FUS has been reported to cause modulation of structural and functional plasticity (changes in dendritic structure, function, and neurotransmitter receptors) in the rat hippocampus *in vitro* (Huang et al., 2019). In anaesthetized rats, transcranial FUS is reported to induce synaptic plasticity in the hippocampus (Niu et al., 2022). Other positive effects on cognition have been reported, through the actions of ultrasound on the BBB. By transiently opening the BBB using scanning ultrasound combined with microbubbles, we restored LTP induction in the dentate gyrus area of the hippocampus in physiologically aged, senescent mice with impaired cognitive function (Blackmore et al., 2021). In 2023, FUS-mediated BBB opening was reported to significantly enhance LTP at Schaffer collaterals of the hippocampus and rescue cognitive dysfunction and working memory in the 5xFAD mouse model of AD (Kong et al., 2023). The FUS-mediated BBB opening enhances adult hippocampal neurogenesis, and the combination of FUS with Aducanumab (a drug used in the treatment of AD), attenuates cognitive function deficits in the same mouse model of AD (Kong et al., 2022).

Particular patterns of neural stimulation, such as theta burst stimulation, applied using FUS, also have directly positive neuromodulatory effects, as they do using localized electrical stimulation (Barry et al., 2014; Boddington et al., 2020). In the recent past, intermittent, and continuous theta burst ultrasound stimulation (TBUS) was reported to induce LTP and LTD respectively, as recorded by changes in motor-evoked potentials in mice (Kim et al., 2024). In this experiment, a theta burst pattern (four pulses at 30-Hz gamma frequency, repeated at 5-Hz theta frequency) was used to deliver 500-KHz US pulses for 17 ms. Intermittent TBUS delivered 2-s theta burst trains that were repeated every 10 s, while continuous TBUS delivered continuous repetitions of the same train. In humans, TBUS can also cause long-lasting alterations in motor cortical excitability (Samuel et al., 2022). Repetitive transcranial ultrasound produces long-lasting effects on motor behavior and motor cortex excitability without any side effects (Zhang et al., 2021). Together, ultrasound-mediated synaptic plasticity may be a mechanism of interest to investigate and potentially elucidate the path for therapeutic approaches targeting PD and other neurological conditions.

## 7 How to elucidate mechanisms of ultrasound-mediated neuromodulation for clinical application

One major advantage of FUS over other therapies for the treatment of basal ganglia disorders is its non-invasiveness and ability to penetrate deeply into brain tissues. The skull presents a significant challenge for accurate ultrasound penetration and targeting due to its varying thickness and density. Technological advancements in transducer design and ultrasound parameters are increasingly improving the ability to target deep brain nuclei for neuromodulation. For further information regarding the current clinical application of ultrasound transducers, please refer to Gandhi et al. (2022).

The use of multielement ultrasound transducers with many independently controllable transducer elements has revolutionized the targeting of deep brain areas. These ultrasound arrays can adjust the phase of each element to constructively interfere with the ultrasound waves at the target site, leading to tighter and more focused energy deposition at deep brain nuclei. This results in a higher intensity at the focal point and better isolation of the target region. In contrast, single-element transducers, which are often employed in hand-held operator-controlled systems (Gandhi et al., 2022), emit a less focused and electronically uncontrollable beam. Phased array transducers can correct for skull and brain variations using adaptive focusing techniques, where the beam is dynamically adjusted to minimize distortions as it passes through different tissues (Hynynen et al., 2004; Clement and Hynynen, 2002; Aubry et al., 2003). This enhances the accuracy of the ultrasound energy reaching deep brain nuclei, minimizing the heating of the scalp and skull where the transducers are placed, by distributing the energy across activated elements and relying on additive effects at the focus.

Thus, technological advances in ultrasound transducer technology have meant that the basal ganglia, seated deep within the brain, can be selectively and non-invasively targeted by FUS for the treatment of neurological disorders such as PD. In the paragraphs below, the various methods that could be used together with ultrasound to investigate the underlying mechanisms activated during FUS treatment have been outlined.

### 7.1 Fiber photometry

Ultrasound treatment lacks the specificity to record the changes in neuronal activation and function, but it can be applied together with other methods. These include fiber photometry and optogenetics, which, with ultrasound, could provide comprehensive and practical information for use in therapeutic approaches, including diagnosis and drug delivery. The preferred method to study neuronal function, due to its good temporal resolution, is offered by electrophysiology, which has restricted use *in vivo*. For example, intracellular recordings are usually limited to studies including a small number of cells (Reynolds et al., 2001; Reynolds et al., 2022), whereas extracellular recordings can measure activity from many cell types at the same time, thereby lacking cellular specificity (Buzsáki et al., 2012). Although fMRI has helped us understand how neural networks that respond to external inputs are dispersed throughout vast brain areas, it has limited spatial and temporal resolution.

To understand the ionic or molecular mechanism triggered by FUS in deep brain tissues, including the basal ganglia, it is imperative to examine these effects at the neuronal level. Combining methods such as fiber photometry or two-photon microscopy with fluorescent reporters (such as calcium indicators) allows for the cellular resolution investigation of neural networks in an intact brain, which is one way to address the lack of spatial information (Sych et al., 2019). Using genetically encoded calcium indicators (GECIs such as GCaMP), cell populations of interest can be targeted, allowing optical monitoring of their activity at cellular and even subcellular precision. Fiber photometry uses a thin optical cannula inserted into the tissue and works well for capturing brain activity at the neuronal population level in freely moving animals. It can also be used to gather signals from various parts of the rodent’s brain simultaneously (Byron and Sakata, 2024). This will enable researchers to address US-mediated effects at the fundamental level within the brain. Thus, integrating fiber photometry with ultrasound could help in mapping the intricate relationships between brain networks and behavior in freely moving animals, providing crucial knowledge of the fundamental processes of ultrasound-mediated neuromodulation in deep brain tissues such as the basal ganglia.

### 7.2 Sonogenetics

Another approach to investigating the mechanisms of ultrasound-mediated neuromodulation is sonogenetics, which uses ultrasound to manipulate and control cells genetically engineered with proteins responsive to ultrasound, in order to systematically alter cellular functions. Bioengineering is often used to fine-tune cellular systems responsive to FUS to boost their effectiveness along with spatial resolution and mitigate off-target effects (Ibsen et al., 2015). The mechanism behind sonogenetics is based on two pillars: first, the insertion of a gene or protein into a cell that responds to ultrasound, and second, the use of genetically engineered proteins or biomolecules to enhance the mechanical effects of FUS on target cells (Gao et al., 2024). One of the most studied sonogenetics mediators is the transient receptor potential (TRP) family of cationic channels, which are involved in sensory physiological processes and have conserved sequences from nematodes to humans (Samanta et al., 2018). TRP channels function as transducers of signals by modifying membrane potential through changes in the level of intracellular Ca^2+^. These channels activate at ultrasonic frequencies lower than 3 MHz (Chu et al., 2022). Interestingly, TRPC1 (TRP complex 1) is downregulated in PD patients (Dietrich et al., 2014). Thus, sonogenetics is an emerging technology that can be effectively applied to manipulate and control cellular functions in superficial as well as deep brain tissues.

There is growing interest in understanding the mechanisms of pathways triggered by using FUS for neuromodulation that could easily affect deep brain regions such as the basal ganglia. In these early days of research on the cellular effects of ultrasound, both fiber photometry and sonogenetics may open new avenues for studying FUS-mediated neuromodulation in brain circuitry and hence may open the pathway to the treatment of basal ganglia-related impairments and disorders.

## 8 Advancements and future directions

### 8.1 Miniaturization and wearable ultrasound devices

Ultrasound is a safe, non-invasive, versatile, and cost-effective method that assists in wound healing and drug delivery to patients under treatment. Wearable ultrasound technology frees the patient from restricted movement and has the potential to significantly enhance individualized medical treatment. With wearable solutions, a single or an array of transducers are arranged in a way to cling firmly to the skin, wirelessly transferring data in real-time to smart devices. Recent developments in wearable ultrasound technology include the imaging of diverse internal organs as a diagnostic and monitoring tool (Wang et al., 2022), and the monitoring of deep tissue signals, including blood pressure, cardiac output, and heart rate, in moving subjects (Lin et al., 2024). Limited miniaturized ultrasound techniques have been utilized in the brain, such as for cerebral blood flow monitoring (Zhou et al., 2024), neuromodulation (Hou et al., 2022), and in a clinical trial of PD patients (Zhong et al., 2024). Miniaturizing wearable ultrasound devices requires further improvement, including stable and reliable connectivity between transducers and skin, increased sensitivity, and specificity over targeting location. Additionally, the transducer size and shape need to be more customisable to better suit the needs of the patient and maximize acoustic targeting.

### 8.2 Multimodal approaches combining ultrasound with other modalities

In the last 5 years, wearable ultrasound has been feasibly integrated with multiple sensing modalities such as chemical sensing (Sempionatto et al., 2021), temperature sensing (Wang et al., 2023), photoacoustic sensing (Gao et al., 2022), and cardiotocography or electronic fetal monitoring (Nguyen et al., 2018). Exciting developments in the field of wearable ultrasound technology are anticipated to enhance healthcare and the quality of life for patients living with diseases related to the basal ganglia and other conditions.

### 8.3 Integration of artificial intelligence in ultrasound imaging and modulation

Although there is also the potential to gather continuous information using wearable transmitting and receiving ultrasound technology, decoding huge quantities of data poses difficulties for interpretation. Because of the complexity and volume of data, it has been proposed that artificial intelligence (AI) is well-placed to examine long-term patterns generated from continuous data to streamline automated data processing, data interpretation, and decision-making (Benke and Benke, 2018). Thus, AI can help enhance wearable ultrasound technology by remotely controlling the device and significantly enhancing the ability to monitor deep brain tissues, such as the basal ganglia, with high accuracy. As a result, in the future, AI integrated with wearable US technology may potentially carry out preliminary diagnoses and provide necessary treatment without involving a physician on the frontlines.

### 8.4 Ultrasonic reporters of Ca^2+^ for deep tissue imaging

In 2023, Jin et al. reported the first dynamic, noninvasive, reversible, allosteric acoustic biosensor named URoC (ultrasound reporter of Ca^2+^) for deep tissue imaging in mammalian cells. Following the mechanisms of fluorescent GECIs, URoC uses engineered gas to undergo a reversible segment conformational change in the presence of calcium, thereby increasing the flexibility of the structures and the nonlinear acoustic characteristics (Jin et al., 2023). They further reported that this sensor has a significant 4.7-fold increase in response to calcium changes *in vitro*. Crucially, it is entirely genetically encoded and functional within mammalian cells, providing greater than 170% contrast in response to increased intracellular calcium. Together, the URoC technique is a promising advancement in the field of deep tissue imaging and has presented researchers with a unique way to investigate calcium signaling in intact living organisms (Jin et al., 2023). Thus, ultrasound can also be employed to track dynamic cellular processes in the natural setting of intact living animals, thereby advancing research into exploring the cell-specific effects of drug action deep inside the brain.

## 9 Conclusion

Focused ultrasound has the potential to revolutionize treatment strategies for the basal ganglia. In addition to diagnosis, ultrasound can also be used for direct neural modulation, drug delivery at precise locations, and monitoring of deep brain tissue changes following drug treatment.

Since patient movement is either limited or impaired in basal ganglia disorders, wearable US technology innovation could be effective for remote treatment based on consultations and teledata delivered from wearable technology. Although wearable and miniaturized FUS technology is in the early stages, further optimization is needed to enhance the sensitivity and specificity of treatment strategies. Future breakthroughs and advances in transducer materials, miniature electronics, AI, and control algorithms are required to address the issues and improve the functionality of the current FUS-based technology, for improved treatment of basal ganglia disorders.

## References

[ref1] Alexander G. E. Delong M. R. Strick P. L. (1986). Parallel organization of functionally segregated circuits linking basal ganglia and cortex. Annu. Rev. Neurosci. 9, 357–381. doi: 10.1146/annurev.ne.09.030186.002041 3085570

[ref2] Aryal M. Vykhodtseva N. Zhang Y. Z. Mcdannold N. (2015). Multiple sessions of liposomal doxorubicin delivery via focused ultrasound mediated blood-brain barrier disruption: a safety study. J. Control. Release 204, 60–69. doi: 10.1016/j.jconrel.2015.02.033, PMID: 25724272 PMC4385501

[ref3] Aubry J. F. Tanter M. (2016). Mr-guided transcranial focused ultrasound. Adv. Exp. Med. Biol. 880, 97–111. doi: 10.1007/978-3-319-22536-4_6 26486334

[ref4] Aubry J. F. Tanter M. Pernot M. Thomas J. L. Fink M. (2003). Experimental demonstration of noninvasive transskull adaptive focusing based on prior computed tomography scans. J. Acoust. Soc. Am. 113, 84–93. doi: 10.1121/1.1529663 12558249

[ref5] Bachu V. S. Kedda J. Suk I. Green J. J. Tyler B. (2021). High-intensity focused ultrasound: a review of mechanisms and clinical applications. Ann. Biomed. Eng. 49, 1975–1991. doi: 10.1007/s10439-021-02833-9, PMID: 34374945 PMC8608284

[ref6] Badran B. W. Caulfield K. A. Stomberg-Firestein S. Summers P. M. Dowdle L. T. Savoca M.. (2020). Sonication of the anterior thalamus with Mri-guided transcranial focused ultrasound (tfus) alters pain thresholds in healthy adults: a double-blind, sham-controlled study. Brain Stimul. 13, 1805–1812. doi: 10.1016/j.brs.2020.10.007, PMID: 33127579 PMC7888561

[ref7] Ballantine H. T. Jr. Bell E. Manlapaz J. (1960). Progress and problems in the neurological applications of focused ultrasound. J. Neurosurg. 17, 858–876. doi: 10.3171/jns.1960.17.5.0858, PMID: 13686380

[ref8] Bangham A. D. Standish M. M. Watkins J. C. (1965). Diffusion of univalent ions across the lamellae of swollen phospholipids. J. Mol. Biol. 13, 238–252. doi: 10.1016/S0022-2836(65)80093-6, PMID: 5859039

[ref9] Banks W. A. (2016). From blood-brain barrier to blood-brain interface: new opportunities for Cns drug delivery. Nat. Rev. Drug Discov. 15, 275–292. doi: 10.1038/nrd.2015.21, PMID: 26794270

[ref10] Barenholz Y. (2001). Liposome application: problems and prospects. Curr. Opin. Colloid Interface Sci. 6, 66–77. doi: 10.1016/S1359-0294(00)00090-X

[ref11] Barry M. D. Boddington L. J. Igelstrom K. M. Gray J. P. Shemmell J. Tseng K. Y.. (2014). Utility of intracerebral theta burst electrical stimulation to attenuate interhemispheric inhibition and to promote motor recovery after cortical injury in an animal model. Exp. Neurol. 261, 258–266. doi: 10.1016/j.expneurol.2014.05.023, PMID: 24905955

[ref12] Barzegar-Fallah A. Gandhi K. Rizwan S. B. Slatter T. L. Reynolds J. N. J. (2022). Harnessing ultrasound for targeting drug delivery to the brain and breaching the blood-brain tumour barrier. Pharmaceutics 15:5. doi: 10.3390/pharmaceutics14102231 PMC9607160 36297666

[ref13] Bedbrook C. N. Deverman B. E. Gradinaru V. (2018). Viral strategies for targeting the central and peripheral nervous systems. Annu. Rev. Neurosci. 41, 323–348. doi: 10.1146/annurev-neuro-080317-062048 29709207

[ref14] Beisteiner R. Matt E. Fan C. Baldysiak H. Schönfeld M. Philippi Novak T.. (2020). Transcranial pulse stimulation with ultrasound in Alzheimer's disease-a new navigated focal brain therapy. Adv. Sci. 7:1902583. doi: 10.1002/advs.201902583, PMID: 32042569 PMC7001626

[ref15] Benke K. Benke G. (2018). Artificial intelligence and big data in public health. Int. J. Environ. Res. Public Health 15:2796. doi: 10.3390/ijerph15122796, PMID: 30544648 PMC6313588

[ref16] Blackmore J. Shrivastava S. Sallet J. Butler C. R. Cleveland R. O. (2019). Ultrasound Neuromodulation: a review of results, mechanisms and safety. Ultrasound Med. Biol. 45, 1509–1536. doi: 10.1016/j.ultrasmedbio.2018.12.015, PMID: 31109842 PMC6996285

[ref17] Blackmore D. G. Turpin F. Palliyaguru T. Evans H. T. Chicoteau A. Lee W.. (2021). Low-intensity ultrasound restores long-term potentiation and memory in senescent mice through pleiotropic mechanisms including Nmdar signaling. Mol. Psychiatry 26, 6975–6991. doi: 10.1038/s41380-021-01129-7, PMID: 34040151 PMC8760044

[ref18] Blesa J. Pineda-Pardo J. A. Inoue K. I. Gasca-Salas C. Balzano T. Del Rey N. L.. (2023). Bbb opening with focused ultrasound in nonhuman primates and Parkinson's disease patients: targeted Aav vector delivery and pet imaging. Sci. Adv. 9:eadf4888. doi: 10.1126/sciadv.adf4888, PMID: 37075119 PMC10115413

[ref19] Bliss T. V. Gardner-Medwin A. R. (1973). Long-lasting potentiation of synaptic transmission in the dentate area of the unanaestetized rabbit following stimulation of the perforant path. J. Physiol. 232, 357–374. doi: 10.1113/jphysiol.1973.sp010274, PMID: 4727085 PMC1350459

[ref20] Boddington L. J. Gray J. P. Schulz J. M. Reynolds J. N. J. (2020). Low-intensity contralesional electrical theta burst stimulation modulates ipsilesional excitability and enhances stroke recovery. Exp. Neurol. 323:113071. doi: 10.1016/j.expneurol.2019.113071, PMID: 31669070

[ref21] Bond A. E. Shah B. B. Huss D. S. Dallapiazza R. F. Warren A. Harrison M. B.. (2017). Safety and efficacy of focused ultrasound thalamotomy for patients with medication-refractory, tremor-dominant Parkinson disease: a randomized clinical trial. JAMA Neurol. 74, 1412–1418. doi: 10.1001/jamaneurol.2017.3098 29084313 PMC5822192

[ref22] Burgess A. Ayala-Grosso C. A. Ganguly M. Jordão J. F. Aubert I. Hynynen K. (2011). Targeted delivery of neural stem cells to the brain using Mri-guided focused ultrasound to disrupt the blood-brain barrier. PLoS One 6:e27877. doi: 10.1371/journal.pone.0027877, PMID: 22114718 PMC3218061

[ref23] Burgess A. Shah K. Hough O. Hynynen K. (2015). Focused ultrasound-mediated drug delivery through the blood-brain barrier. Expert. Rev. Neurother. 15, 477–491. doi: 10.1586/14737175.2015.1028369, PMID: 25936845 PMC4702264

[ref24] Buzsáki G. Anastassiou C. A. Koch C. (2012). The origin of extracellular fields and currents — EEG, ECoG, LFP and spikes. Nat. Rev. Neurosci. 13, 407–420. doi: 10.1038/nrn3241, PMID: 22595786 PMC4907333

[ref25] Byron N. Sakata S. (2024). Fiber photometry-based investigation of brain function and dysfunction. Neurophotonics 11:S11502. doi: 10.1117/1.NPh.11.S1.S11502 38077295 PMC10704183

[ref26] Carpentier A. Canney M. Vignot A. Reina V. Beccaria K. Horodyckid C.. (2016). Clinical trial of blood-brain barrier disruption by pulsed ultrasound. Sci. Transl. Med. 8:343re2. doi: 10.1126/scitranslmed.aaf6086 27306666

[ref27] Chang W. S. Jung H. H. Zadicario E. Rachmilevitch I. Tlusty T. Vitek S.. (2016). Factors associated with successful magnetic resonance-guided focused ultrasound treatment: efficiency of acoustic energy delivery through the skull. J. Neurosurg. 124, 411–416. doi: 10.3171/2015.3.JNS142592, PMID: 26361280

[ref28] Chen K. T. Lin Y. J. Chai W. Y. Lin C. J. Chen P. Y. Huang C. Y.. (2020a). Neuronavigation-guided focused ultrasound (Navifus) for transcranial blood-brain barrier opening in recurrent glioblastoma patients: clinical trial protocol. Ann. Transl. Med. 8:673. doi: 10.21037/atm-20-344, PMID: 32617293 PMC7327352

[ref29] Chen J. Liu J. Chen W. Shang D. Zhang Q. Li Y.. (2024). Skin-conformable flexible and stretchable ultrasound transducer for wearable imaging. IEEE Trans. Ultrason. Ferroelectr. Freq. Control 71, 811–820. doi: 10.1109/TUFFC.2024.3352655, PMID: 38206777

[ref30] Chen S. G. Tsai C. H. Lin C. J. Lee C. C. Yu H. Y. Hsieh T. H.. (2020b). Transcranial focused ultrasound pulsation suppresses pentylenetetrazol induced epilepsy in vivo. Brain Stimul. 13, 35–46. doi: 10.1016/j.brs.2019.09.011, PMID: 31575487

[ref31] Cheng Z. Li M. Dey R. Chen Y. (2021). Nanomaterials for cancer therapy: current progress and perspectives. J. Hematol. Oncol. 14:85. doi: 10.1186/s13045-021-01096-0, PMID: 34059100 PMC8165984

[ref32] Christine C. W. Bankiewicz K. S. Van Laar A. D. Richardson R. M. Ravina B. Kells A. P.. (2019). Magnetic resonance imaging-guided phase 1 trial of putaminal Aadc gene therapy for Parkinson's disease. Ann. Neurol. 85, 704–714. doi: 10.1002/ana.25450, PMID: 30802998 PMC6593762

[ref33] Christine C. W. Richardson R. M. Van Laar A. D. Thompson M. E. Fine E. M. Khwaja O. S.. (2022). Safety of Aadc gene therapy for moderately advanced Parkinson disease: three-year outcomes from the Pd-1101 trial. Neurology 98, e40–e50. doi: 10.1212/WNL.0000000000012952, PMID: 34649873 PMC8726573

[ref34] Chu Y. C. Lim J. Chien A. Chen C. C. Wang J. L. (2022). Activation of mechanosensitive ion channels by ultrasound. Ultrasound Med. Biol. 48, 1981–1994. doi: 10.1016/j.ultrasmedbio.2022.06.008 35945063

[ref35] Clement G. T. Hynynen K. (2002). A non-invasive method for focusing ultrasound through the human skull. Phys. Med. Biol. 47, 1219–1236. doi: 10.1088/0031-9155/47/8/301 12030552

[ref36] Coluccia D. Fandino J. Schwyzer L. O’gorman R. Remonda L. Anon J.. (2014). First noninvasive thermal ablation of a brain tumor with Mr-guided focused ultrasound. J. Ther. Ultrasound 2:17. doi: 10.1186/2050-5736-2-17, PMID: 25671132 PMC4322509

[ref37] Dallapiazza R. F. Timbie K. F. Holmberg S. Gatesman J. Lopes M. B. Price R. J.. (2018). Noninvasive neuromodulation and thalamic mapping with low-intensity focused ultrasound. J. Neurosurg. 128, 875–884. doi: 10.3171/2016.11.JNS16976, PMID: 28430035 PMC7032074

[ref38] Dauer W. Przedborski S. (2003). Parkinson's disease: mechanisms and models. Neuron 39, 889–909. doi: 10.1016/S0896-6273(03)00568-3 12971891

[ref39] Dayton P. Klibanov A. Brandenburger G. Ferrara K. (1999). Acoustic radiation force in vivo: a mechanism to assist targeting of microbubbles. Ultrasound Med. Biol. 25, 1195–1201. doi: 10.1016/S0301-5629(99)00062-9, PMID: 10576262

[ref40] Deffieux T. Younan Y. Wattiez N. Tanter M. Pouget P. Aubry J. F. (2013). Low-intensity focused ultrasound modulates monkey visuomotor behavior. Curr. Biol. 23, 2430–2433. doi: 10.1016/j.cub.2013.10.029, PMID: 24239121

[ref41] Dietrich A. Fahlbusch M. Gudermann T. (2014). Classical transient receptor potential 1 (Trpc1): channel or channel regulator? Cells 3, 939–962. doi: 10.3390/cells3040939, PMID: 25268281 PMC4276908

[ref42] Dos Santos Rodrigues B. Banerjee A. Kanekiyo T. Singh J. (2019). Functionalized liposomal nanoparticles for efficient gene delivery system to neuronal cell transfection. Int. J. Pharm. 566, 717–730. doi: 10.1016/j.ijpharm.2019.06.026, PMID: 31202901 PMC6671319

[ref43] Dréan A. Lemaire N. Bouchoux G. Goldwirt L. Canney M. Goli L.. (2019). Temporary blood-brain barrier disruption by low intensity pulsed ultrasound increases carboplatin delivery and efficacy in preclinical models of glioblastoma. J. Neuro-Oncol. 144, 33–41. doi: 10.1007/s11060-019-03204-0 31197598

[ref44] Elias W. J. Huss D. Voss T. Loomba J. Khaled M. Zadicario E.. (2013). A pilot study of focused ultrasound thalamotomy for essential tremor. N. Engl. J. Med. 369, 640–648. doi: 10.1056/NEJMoa1300962, PMID: 23944301

[ref45] Elias W. J. Lipsman N. Ondo W. G. Ghanouni P. Kim Y. G. Lee W.. (2016). A randomized trial of focused ultrasound thalamotomy for essential tremor. N. Engl. J. Med. 375, 730–739. doi: 10.1056/NEJMoa1600159, PMID: 27557301

[ref46] Fry F. J. Ades H. W. Fry W. J. (1958). Production of reversible changes in the central nervous system by ultrasound. Science 127, 83–84, PMID: 13495483 10.1126/science.127.3289.83

[ref47] Gandhi K. Barzegar-Fallah A. Banstola A. Rizwan S. B. Reynolds J. N. J. (2022). Correction: Gandhi et al. ultrasound-mediated blood-brain barrier disruption for drug delivery: a systematic review of protocols, efficacy, and safety outcomes from preclinical and clinical studies. Pharmaceutics 14:833. doi: 10.3390/pharmaceutics15010005 35456667 PMC9029131

[ref48] Gao X. Chen X. Hu H. Wang X. Yue W. Mu J.. (2022). A photoacoustic patch for three-dimensional imaging of hemoglobin and core temperature. Nat. Commun. 13:7757. doi: 10.1038/s41467-022-35455-3, PMID: 36522334 PMC9755152

[ref49] Gao T. Niu L. Wu X. Dai D. Zhou Y. Liu M.. (2024). Sonogenetics-controlled synthetic designer cells for cancer therapy in tumor mouse models. Cell Rep. Med. 5:101513. doi: 10.1016/j.xcrm.2024.101513, PMID: 38608697 PMC11148564

[ref50] Gibbs V. Cole D. Sassano A. (2009). Ultrasound Physics and Technology: How, why and when. United Kingdom: Churchill Livingstone/Elsevier.

[ref51] Gourevich D. Dogadkin O. Volovick A. Wang L. Gnaim J. Cochran S.. (2013). Ultrasound-mediated targeted drug delivery with a novel cyclodextrin-based drug carrier by mechanical and thermal mechanisms. J. Control. Release 170, 316–324. doi: 10.1016/j.jconrel.2013.05.038, PMID: 23770006

[ref52] Guo H. Salahshoor H. Wu D. Yoo S. Sato T. Tsao D. Y.. (2023). Effects of focused ultrasound in a "clean" mouse model of ultrasonic neuromodulation. iScience 26:108372. doi: 10.1016/j.isci.2023.108372, PMID: 38047084 PMC10690554

[ref53] Haar G. T. Coussios C. (2007). High intensity focused ultrasound: past, present and future. Int. J. Hyperth. 23, 85–87. doi: 10.1080/02656730601185924 17578334

[ref54] Hameroff S. Trakas M. Duffield C. Annabi E. Gerace M. B. Boyle P.. (2013). Transcranial ultrasound (Tus) effects on mental states: a pilot study. Brain Stimul. 6, 409–415. doi: 10.1016/j.brs.2012.05.002, PMID: 22664271

[ref55] Heimburg T. (2012). The capacitance and electromechanical coupling of lipid membranes close to transitions: the effect of electrostriction. Biophys. J. 103, 918–929. doi: 10.1016/j.bpj.2012.07.010, PMID: 23009841 PMC3433620

[ref56] Heiss J. D. Lungu C. Hammoud D. A. Herscovitch P. Ehrlich D. J. Argersinger D. P.. (2019). Trial of magnetic resonance-guided putaminal gene therapy for advanced Parkinson's disease. Mov. Disord. 34, 1073–1078. doi: 10.1002/mds.27724, PMID: 31145831 PMC6642028

[ref57] Higuchi Y. Matsuda S. Serizawa T. (2017). Gamma knife radiosurgery in movement disorders: indications and limitations. Mov. Disord. 32, 28–35. doi: 10.1002/mds.26625 27029223

[ref58] Hinow P. Radunskaya A. Mackay S. M. Reynolds J. N. Schroeder M. Tan E. W.. (2016). Signaled drug delivery and transport across the blood-brain barrier. J. Liposome Res. 26, 233–245. doi: 10.3109/08982104.2015.1102277 26572864

[ref59] Hou C. Wu Y. Fei C. Qiu Z. Li Z. Sun X.. (2022). An optimized miniaturized ultrasound transducer for transcranial neuromodulation. Front. Neurosci. 16:893108. doi: 10.3389/fnins.2022.893108, PMID: 35801172 PMC9253503

[ref60] Huang X. Lin Z. Wang K. Liu X. Zhou W. Meng L.. (2019). Transcranial low-intensity pulsed ultrasound modulates structural and functional synaptic plasticity in rat hippocampus. IEEE Trans. Ultrason. Ferroelectr. Freq. Control 66, 930–938. doi: 10.1109/TUFFC.2019.2903896 30869615

[ref61] Huang S. L. Macdonald R. C. (2004). Acoustically active liposomes for drug encapsulation and ultrasound-triggered release. Biochim. Biophys. Acta 1665, 134–141. doi: 10.1016/j.bbamem.2004.07.003 15471579

[ref62] Hynynen K. Clement G. T. Mcdannold N. Vykhodtseva N. King R. White P. J.. (2004). 500-element ultrasound phased array system for noninvasive focal surgery of the brain: a preliminary rabbit study with ex vivo human skulls. Magn. Reson. Med. 52, 100–107. doi: 10.1002/mrm.20118, PMID: 15236372

[ref63] Hynynen K. Mcdannold N. Clement G. Jolesz F. A. Zadicario E. Killiany R.. (2006). Pre-clinical testing of a phased array ultrasound system for MRI-guided noninvasive surgery of the brain—a primate study. Eur. J. Radiol. 59, 149–156. doi: 10.1016/j.ejrad.2006.04.007, PMID: 16716552

[ref64] Hynynen K. Mcdannold N. Vykhodtseva N. Jolesz F. A. (2001). Noninvasive Mr imaging-guided focal opening of the blood-brain barrier in rabbits. Radiology 220, 640–646. doi: 10.1148/radiol.2202001804, PMID: 11526261

[ref65] Ibsen S. Tong A. Schutt C. Esener S. Chalasani S. H. (2015). Sonogenetics is a non-invasive approach to activating neurons in *Caenorhabditis elegans*. Nat. Commun. 6:8264. doi: 10.1038/ncomms9264, PMID: 26372413 PMC4571289

[ref66] Jin Z. Lakshmanan A. Zhang R. Tran T. A. Rabut C. Dutka P.. (2023). Ultrasonic reporters of calcium for deep tissue imaging of cellular signals. bioRxiv [Preprint]. doi: 10.1101/2023.11.09.566364v1

[ref67] Jung H. H. Kim S. J. Roh D. Chang J. G. Chang W. S. Kweon E. J.. (2015). Bilateral thermal capsulotomy with Mr-guided focused ultrasound for patients with treatment-refractory obsessive-compulsive disorder: a proof-of-concept study. Mol. Psychiatry 20, 1205–1211. doi: 10.1038/mp.2014.154, PMID: 25421403

[ref68] Jung N. Y. Park C. K. Kim M. Lee P. H. Sohn Y. H. Chang J. W. (2018). The efficacy and limits of magnetic resonance-guided focused ultrasound pallidotomy for Parkinson's disease: a phase I clinical trial. J. Neurosurg. 130, 1853–1861. 30095337 10.3171/2018.2.JNS172514

[ref69] Kaplitt M. G. Feigin A. Tang C. Fitzsimons H. L. Mattis P. Lawlor P. A.. (2007). Safety and tolerability of gene therapy with an adeno-associated virus (Aav) borne gad gene for Parkinson's disease: an open label, phase I trial. Lancet 369, 2097–2105. doi: 10.1016/S0140-6736(07)60982-9, PMID: 17586305

[ref70] Kim H. Chiu A. Lee S. D. Fischer K. Yoo S. S. (2014). Focused ultrasound-mediated non-invasive brain stimulation: examination of sonication parameters. Brain Stimul. 7, 748–756. doi: 10.1016/j.brs.2014.06.011, PMID: 25088462 PMC4167941

[ref71] Kim M. Kim C. H. Jung H. H. Kim S. J. Chang J. W. (2018). Treatment of major depressive disorder via magnetic resonance-guided focused ultrasound surgery. Biol. Psychiatry 83, e17–e18. doi: 10.1016/j.biopsych.2017.05.008 28601192

[ref72] Kim Y. S. Ko M. J. Moon H. Sim W. Cho A. S. Gil G.. (2022). Ultrasound-responsive liposomes for targeted drug delivery combined with focused ultrasound. Pharmaceutics 14:1314. doi: 10.3390/pharmaceutics14071314 PMC9315635 35890210

[ref73] Kim K. Lee J. Park M. H. (2023). Microbubble delivery platform for ultrasound-mediated therapy in brain cancers. Pharmaceutics 15:698. doi: 10.3390/pharmaceutics15020698 PMC9959315 36840020

[ref74] Kim H. J. Phan T. T. Lee K. Kim J. S. Lee S. Y. Lee J. M.. (2024). Long-lasting forms of plasticity through patterned ultrasound-induced brainwave entrainment. Sci. Adv. 10:eadk3198. doi: 10.1126/sciadv.adk3198, PMID: 38394205 PMC10889366

[ref75] Kim H. B. Swanberg K. M. Han H. S. Kim J. C. Kim J. W. Lee S.. (2017). Prolonged stimulation with low-intensity ultrasound induces delayed increases in spontaneous hippocampal culture spiking activity. J. Neurosci. Res. 95, 885–896. doi: 10.1002/jnr.23845 27465511

[ref76] King R. L. Brown J. R. Pauly K. B. (2014). Localization of ultrasound-induced in vivo neurostimulation in the mouse model. Ultrasound Med. Biol. 40, 1512–1522. doi: 10.1016/j.ultrasmedbio.2014.01.020, PMID: 24642220

[ref77] Kobus T. Zervantonakis I. K. Zhang Y. Mcdannold N. J. (2016). Growth inhibition in a brain metastasis model by antibody delivery using focused ultrasound-mediated blood-brain barrier disruption. J. Control. Release 238, 281–288. doi: 10.1016/j.jconrel.2016.08.001, PMID: 27496633 PMC5014601

[ref78] Kong C. Ahn J. W. Kim S. Park J. Y. Na Y. C. Chang J. W.. (2023). Long-lasting restoration of memory function and hippocampal synaptic plasticity by focused ultrasound in Alzheimer's disease. Brain Stimul. 16, 857–866. doi: 10.1016/j.brs.2023.05.014, PMID: 37211337

[ref79] Kong C. Yang E. J. Shin J. Park J. Kim S. H. Park S. W.. (2022). Enhanced delivery of a low dose of aducanumab via Fus in 5×fad mice, an ad model. Transl Neurodegener. 11:57. doi: 10.1186/s40035-022-00333-x, PMID: 36575534 PMC9793531

[ref80] Kotulska K. Fattal-Valevski A. Haberlova J. (2021). Recombinant adeno-associated virus serotype 9 gene therapy in spinal muscular atrophy. Front. Neurol. 12:726468. doi: 10.3389/fneur.2021.726468, PMID: 34721262 PMC8548432

[ref81] Kovacs Z. Werner B. Rassi A. Sass J. O. Martin-Fiori E. Bernasconi M. (2014). Prolonged survival upon ultrasound-enhanced doxorubicin delivery in two syngenic glioblastoma mouse models. J. Control. Release 187, 74–82. doi: 10.1016/j.jconrel.2014.05.033 24878186

[ref82] Kubanek J. Shi J. Marsh J. Chen D. Deng C. Cui J. (2016). Ultrasound modulates ion channel currents. Sci. Rep. 6:24170. doi: 10.1038/srep24170, PMID: 27112990 PMC4845013

[ref83] Kusuyama J. Bandow K. Shamoto M. Kakimoto K. Ohnishi T. Matsuguchi T. (2014). Low intensity pulsed ultrasound (Lipus) influences the multilineage differentiation of mesenchymal stem and progenitor cell lines through Rock-cot/Tpl2-Mek-Erk signaling pathway. J. Biol. Chem. 289, 10330–10344. doi: 10.1074/jbc.M113.546382, PMID: 24550383 PMC4036157

[ref84] Lanciego J. L. Luquin N. Obeso J. A. (2012). Functional neuroanatomy of the basal ganglia. Cold Spring Harb. Perspect. Med. 2:a009621. doi: 10.1101/cshperspect.a009621 23071379 PMC3543080

[ref85] Legon W. Bansal P. Tyshynsky R. Ai L. Mueller J. K. (2018). Transcranial focused ultrasound neuromodulation of the human primary motor cortex. Sci. Rep. 8:10007. doi: 10.1038/s41598-018-28320-1, PMID: 29968768 PMC6030101

[ref86] Legon W. Rowlands A. Opitz A. Sato T. F. Tyler W. J. (2012). Pulsed ultrasound differentially stimulates somatosensory circuits in humans as indicated by Eeg and Fmri. PLoS One 7:e51177. doi: 10.1371/journal.pone.0051177, PMID: 23226567 PMC3514181

[ref87] Leksell L. (1956). Echo-encephalography. I. Detection of intracranial complications following head injury. Acta Chir. Scand. 110, 301–315, PMID: 13292078

[ref88] Lewitt P. A. (2015). Levodopa therapy for Parkinson's disease: pharmacokinetics and pharmacodynamics. Mov. Disord. 30, 64–72. doi: 10.1002/mds.26082, PMID: 25449210

[ref89] Li G. Qiu W. Zhang Z. Jiang Q. Su M. Cai R.. (2019). Noninvasive ultrasonic neuromodulation in freely moving mice. IEEE Trans. Biomed. Eng. 66, 217–224. doi: 10.1109/TBME.2018.2821201, PMID: 29993389

[ref90] Li C. Samulski R. J. (2020). Engineering adeno-associated virus vectors for gene therapy. Nat. Rev. Genet. 21, 255–272. doi: 10.1038/s41576-019-0205-4 32042148

[ref91] Lin W. T. Chen R. C. Lu W. W. Liu S. H. Yang F. Y. (2015). Protective effects of low-intensity pulsed ultrasound on aluminum-induced cerebral damage in Alzheimer's disease rat model. Sci. Rep. 5:9671. 25873429 10.1038/srep09671 PMC4397698

[ref92] Lin Z. Meng L. Zou J. Zhou W. Huang X. Xue S.. (2020). Non-invasive ultrasonic neuromodulation of neuronal excitability for treatment of epilepsy. Theranostics 10, 5514–5526. doi: 10.7150/thno.40520, PMID: 32373225 PMC7196311

[ref93] Lin H.-Y. Thomas J. L. (2003). Peg−lipids and oligo(ethylene glycol) surfactants enhance the ultrasonic permeabilizability of liposomes. Langmuir 19, 1098–1105. doi: 10.1021/la026604t

[ref94] Lin M. Zhang Z. Gao X. Bian Y. Wu R. S. Park G.. (2024). A fully integrated wearable ultrasound system to monitor deep tissues in moving subjects. Nat. Biotechnol. 42, 448–457. doi: 10.1038/s41587-023-01800-0, PMID: 37217752

[ref95] Ling L. Wei T. He L. Wang Y. Wang Y. Feng X.. (2017). Low-intensity pulsed ultrasound activates Erk1/2 and Pi3K-Akt signalling pathways and promotes the proliferation of human amnion-derived mesenchymal stem cells. Cell Prolif. 50:e12383. doi: 10.1111/cpr.12383, PMID: 28940899 PMC6529069

[ref96] Lipsman N. Schwartz M. L. Huang Y. Lee L. Sankar T. Chapman M.. (2013). Mr-guided focused ultrasound thalamotomy for essential tremor: a proof-of-concept study. Lancet Neurol. 12, 462–468. doi: 10.1016/S1474-4422(13)70048-6, PMID: 23523144

[ref97] Liu S. H. Lai Y. L. Chen B. L. Yang F. Y. (2017). Ultrasound enhances the expression of brain-derived neurotrophic factor in astrocyte through activation of TrkB-Akt and calcium-Camk signaling pathways. Cereb. Cortex 27, 3152–3160. doi: 10.1093/cercor/bhw169, PMID: 27252349

[ref98] Liu C. Yu K. Niu X. He B. (2021). Transcranial focused ultrasound enhances sensory discrimination capability through somatosensory cortical excitation. Ultrasound Med. Biol. 47, 1356–1366. doi: 10.1016/j.ultrasmedbio.2021.01.025, PMID: 33622622 PMC8011531

[ref99] Luan S. Williams I. Nikolic K. Constandinou T. G. (2014). Neuromodulation: present and emerging methods. Front. Neuroeng. 7:27. doi: 10.3389/fneng.2014.00027 25076887 PMC4097946

[ref100] Mackay S. M. Myint D. M. Easingwood R. A. Hegh D. Y. Wickens J. R. Hyland B. I.. (2019). Dynamic control of neurochemical release with ultrasonically-sensitive nanoshell-tethered liposomes. Commun. Chem. 2:122. doi: 10.1038/s42004-019-0226-0

[ref101] Magrinelli F. Picelli A. Tocco P. Federico A. Roncari L. Smania N.. (2016). Pathophysiology of motor dysfunction in Parkinson's disease as the rationale for drug treatment and rehabilitation. Parkinsons Dis. 2016:9832839. doi: 10.1155/2016/9832839 27366343 PMC4913065

[ref102] Maguire A. M. Bennett J. Aleman E. M. Leroy B. P. Aleman T. S. (2021). Clinical perspective: treating Rpe65-associated retinal dystrophy. Mol. Ther. 29, 442–463. doi: 10.1016/j.ymthe.2020.11.029, PMID: 33278565 PMC7854308

[ref103] Mainprize T. Lipsman N. Huang Y. Meng Y. Bethune A. Ironside S.. (2019). Blood-brain barrier opening in primary brain tumors with non-invasive Mr-guided focused ultrasound: a clinical safety and feasibility study. Sci. Rep. 9:321. doi: 10.1038/s41598-018-36340-0 30674905 PMC6344541

[ref104] Malietzis G. Monzon L. Hand J. Wasan H. Leen E. Abel M.. (2013). High-intensity focused ultrasound: advances in technology and experimental trials support enhanced utility of focused ultrasound surgery in oncology. Br. J. Radiol. 86:20130044. doi: 10.1259/bjr.20130044, PMID: 23403455 PMC3635791

[ref105] Martínez-Fernández R. Rodríguez-Rojas R. Del Álamo M. Hernández-Fernández F. Pineda-Pardo J. A. Dileone M.. (2018). Focused ultrasound subthalamotomy in patients with asymmetric Parkinson's disease: a pilot study. Lancet Neurol. 17, 54–63. doi: 10.1016/S1474-4422(17)30403-9, PMID: 29203153

[ref106] Maruyama M. Tojo H. Toi K. Ienaka Y. Hyodo K. Kikuchi H.. (2022). Effect of doxorubicin release rate from polyethylene glycol-modified liposome on anti-tumor activity in B16-Bl6 tumor-bearing mice. J. Pharm. Sci. 111, 293–297. doi: 10.1016/j.xphs.2021.11.020, PMID: 34861247

[ref107] Mason T. J. (2011). Therapeutic ultrasound an overview. Ultrason. Sonochem. 18, 847–852. doi: 10.1016/j.ultsonch.2011.01.004 21316286

[ref108] Mathai A. Smith Y. (2011). The corticostriatal and corticosubthalamic pathways: two entries, one target. So what? Front. Syst. Neurosci. 5:64. doi: 10.3389/fnsys.2011.00064 21866224 PMC3149683

[ref109] Mcmahon D. Hynynen K. (2017). Acute inflammatory response following increased blood-brain barrier permeability induced by focused ultrasound is dependent on microbubble dose. Theranostics 7, 3989–4000. doi: 10.7150/thno.21630, PMID: 29109793 PMC5667420

[ref110] Mei J. Cheng Y. Song Y. Yang Y. Wang F. Liu Y.. (2009). Experimental study on targeted methotrexate delivery to the rabbit brain via magnetic resonance imaging-guided focused ultrasound. J. Ultrasound Med. 28, 871–880. doi: 10.7863/jum.2009.28.7.871, PMID: 19546329

[ref111] Meyers R. Fry W. J. Fry F. J. Dreyer L. L. Schultz D. F. Noyes R. F. (1959). Early experiences with ultrasonic irradiation of the pallidofugal and nigral complexes in hyperkinetic and hypertonic disorders. J. Neurosurg. 16, 32–54. doi: 10.3171/jns.1959.16.1.0032, PMID: 13621264

[ref112] Miller D. L. Smith N. B. Bailey M. R. Czarnota G. J. Hynynen K. Makin I. R. (2012). Overview of therapeutic ultrasound applications and safety considerations. J. Ultrasound Med. 31, 623–634. doi: 10.7863/jum.2012.31.4.623, PMID: 22441920 PMC3810427

[ref113] Min B. K. Bystritsky A. Jung K. I. Fischer K. Zhang Y. Maeng L. S.. (2011). Focused ultrasound-mediated suppression of chemically-induced acute epileptic Eeg activity. BMC Neurosci. 12:23. doi: 10.1186/1471-2202-12-23, PMID: 21375781 PMC3061951

[ref114] Monti M. M. Schnakers C. Korb A. S. Bystritsky A. Vespa P. M. (2016). Non-invasive ultrasonic thalamic stimulation in disorders of consciousness after severe brain injury: a first-in-man report. Brain Stimul. 9, 940–941. doi: 10.1016/j.brs.2016.07.008, PMID: 27567470

[ref115] Moore M. E. Loft J. M. Clegern W. C. Wisor J. P. (2015). Manipulating neuronal activity in the mouse brain with ultrasound: a comparison with optogenetic activation of the cerebral cortex. Neurosci. Lett. 604, 183–187. doi: 10.1016/j.neulet.2015.07.024, PMID: 26222259 PMC6071669

[ref116] Morris R. G. Anderson E. Lynch G. S. Baudry M. (1986). Selective impairment of learning and blockade of long-term potentiation by an N-methyl-D-aspartate receptor antagonist, Ap5. Nature 319, 774–776. doi: 10.1038/319774a0, PMID: 2869411

[ref117] Mouratidis P. X. E. Rivens I. Civale J. Symonds-Tayler R. Ter Haar G. (2019). Relationship between thermal dose and cell death for "rapid" ablative and "slow" hyperthermic heating. Int. J. Hyperth. 36, 229–243. doi: 10.1080/02656736.2018.1558289, PMID: 30700171

[ref118] Mujoo H. Reynolds J. N. Tucker I. G. (2015). The influence of bile salts on the response of liposomes to ultrasound. J. Liposome Res. 26, 87–95. doi: 10.3109/08982104.2015.1019515 25826202

[ref119] Muramatsu S. Fujimoto K. Kato S. Mizukami H. Asari S. Ikeguchi K.. (2010). A phase I study of aromatic L-amino acid decarboxylase gene therapy for Parkinson's disease. Mol. Ther. 18, 1731–1735. doi: 10.1038/mt.2010.135, PMID: 20606642 PMC2956925

[ref120] Nakano T. Rizwan S. B. Myint D. M. A. Gray J. Mackay S. M. Harris P.. (2022). An on-demand drug delivery system for control of epileptiform seizures. Pharmaceutics 14:468. doi: 10.3390/pharmaceutics14020468 PMC8879600 35214199

[ref121] Nelson E. Lindstrom P. A. Haymaker W. (1959). Pathological effects of ultrasound on the human brain. A study of 25 cases in which ultrasonic irradiation was used as a lobotomy procedure. J. Neuropathol. Exp. Neurol. 18, 489–508. doi: 10.1097/00005072-195910000-00001 14426446

[ref122] Nguyen K. Bamgbose E. Cox B. P. Huang S. P. Mierzwa A. Hutchins S.. (2018). Wearable fetal monitoring solution for improved mobility during labor & delivery. Annu. Int. Conf. IEEE Eng. Med. Biol. Soc. 2018, 4397–4400. doi: 10.1109/EMBC.2018.8513321, PMID: 30441327

[ref123] Nicodemus N. E. Becerra S. Kuhn T. P. Packham H. R. Duncan J. Mahdavi K.. (2019). Focused transcranial ultrasound for treatment of neurodegenerative dementia. Alzheimers Dement. 5, 374–381. doi: 10.1016/j.trci.2019.06.007, PMID: 31440580 PMC6700262

[ref124] Niethammer M. Tang C. C. Lewitt P. A. Rezai A. R. Leehey M. A. Ojemann S. G.. (2017). Long-term follow-up of a randomized Aav2-gad gene therapy trial for Parkinson's disease. JCI Insight 2:e90133. doi: 10.1172/jci.insight.90133, PMID: 28405611 PMC5374069

[ref125] Niu X. Yu K. He B. (2022). Transcranial focused ultrasound induces sustained synaptic plasticity in rat hippocampus. Brain Stimul. 15, 352–359. doi: 10.1016/j.brs.2022.01.015, PMID: 35104664 PMC9295311

[ref126] Nowicki A. (2020). Safety of ultrasonic examinations; thermal and mechanical indices. Med. Ultrasound 22, 203–210. doi: 10.11152/mu-2372 32399527

[ref127] O’brien W. D. Jr. (2007). Ultrasound-biophysics mechanisms. Prog. Biophys. Mol. Biol. 93, 212–255. doi: 10.1016/j.pbiomolbio.2006.07.010, PMID: 16934858 PMC1995002

[ref128] Obermeier B. Daneman R. Ransohoff R. M. (2013). Development, maintenance and disruption of the blood-brain barrier. Nat. Med. 19, 1584–1596. doi: 10.1038/nm.3407, PMID: 24309662 PMC4080800

[ref129] Pardridge W. M. (2012). Drug transport across the blood-brain barrier. J. Cereb. Blood Flow Metab. 32, 1959–1972. doi: 10.1038/jcbfm.2012.126, PMID: 22929442 PMC3494002

[ref130] Parodi A. Rudzińska M. Deviatkin A. A. Soond S. M. Baldin A. V. Zamyatnin A. A. Jr. (2019). Established and emerging strategies for drug delivery across the blood-brain barrier in brain cancer. Pharmaceutics 11:245. doi: 10.3390/pharmaceutics11050245, PMID: 31137689 PMC6572140

[ref131] Pelekanos M. Leinenga G. Odabaee M. Odabaee M. Saifzadeh S. Steck R.. (2018). Establishing sheep as an experimental species to validate ultrasound-mediated blood-brain barrier opening for potential therapeutic interventions. Theranostics 8, 2583–2602. doi: 10.7150/thno.22852, PMID: 29721100 PMC5928910

[ref132] Perozo E. Kloda A. Cortes D. M. Martinac B. (2002). Physical principles underlying the transduction of bilayer deformation forces during mechanosensitive channel gating. Nat. Struct. Biol. 9, 696–703. doi: 10.1038/nsb827 12172537

[ref133] Petrov A. G. (2002). Flexoelectricity of model and living membranes. Biochim. Biophys. Acta 1561, 1–25. doi: 10.1016/S0304-4157(01)00007-7, PMID: 11988178

[ref134] Plaksin M. Kimmel E. Shoham S. (2016). Cell-type-selective effects of intramembrane cavitation as a unifying theoretical framework for ultrasonic neuromodulation. eNeuro 3:ENEURO.0136-15.2016. 10.1523/ENEURO.0136-15.2016 PMC4917736 27390775

[ref135] Prieto M. L. Ömer O. Khuri-Yakub B. T. Maduke M. C. (2013). Dynamic response of model lipid membranes to ultrasonic radiation force. PLoS One 8:e77115. doi: 10.1371/journal.pone.0077115, PMID: 24194863 PMC3806737

[ref136] Redgrave P. Vautrelle N. Reynolds J. N. (2011). Functional properties of the basal ganglia's re-entrant loop architecture: selection and reinforcement. Neuroscience 198, 138–151. doi: 10.1016/j.neuroscience.2011.07.060 21821101

[ref137] Regenold M. Bannigan P. Evans J. C. Waspe A. Temple M. J. Allen C. (2022). Turning down the heat: the case for mild hyperthermia and thermosensitive liposomes. Nanomedicine 40:102484. doi: 10.1016/j.nano.2021.102484, PMID: 34748961

[ref138] Reynolds J. N. J. Avvisati R. Dodson P. D. Fisher S. D. Oswald M. J. Wickens J. R.. (2022). Coincidence of cholinergic pauses, dopaminergic activation and depolarisation of spiny projection neurons drives synaptic plasticity in the striatum. Nat. Commun. 13:1296. doi: 10.1038/s41467-022-28950-0, PMID: 35277506 PMC8917208

[ref139] Reynolds J. N. J. Hyland B. I. Wickens J. R. (2001). A cellular mechanism of reward-related learning. Nature 413, 67–70. doi: 10.1038/35092560 11544526

[ref140] Samanta A. Hughes T. E. T. Moiseenkova-Bell V. Y. (2018). Transient receptor potential (Trp) channels. Subcell. Biochem. 87, 141–165. doi: 10.1007/978-981-10-7757-9_6, PMID: 29464560 PMC6038138

[ref141] Samuel N. Zeng K. Harmsen I. E. Ding M. Y. R. Darmani G. Sarica C.. (2022). Multi-modal investigation of transcranial ultrasound-induced neuroplasticity of the human motor cortex. Brain Stimul. 15, 1337–1347. doi: 10.1016/j.brs.2022.10.001 36228977

[ref142] Sapareto S. A. Dewey W. C. (1984). Thermal dose determination in cancer therapy. Int. J. Radiat. Oncol. Biol. Phys. 10, 787–800. doi: 10.1016/0360-3016(84)90379-1 6547421

[ref143] Schreglmann S. R. Krauss J. K. Chang J. W. Bhatia K. P. Kägi G. (2018). Functional lesional neurosurgery for tremor: a systematic review and meta-analysis. J. Neurol. Neurosurg. Psychiatry 89, 717–726. doi: 10.1136/jnnp-2017-316302 29326290

[ref144] Seeger H. M. Aldrovandi L. Alessandrini A. Facci P. (2010). Changes in single K(+) channel behavior induced by a lipid phase transition. Biophys. J. 99, 3675–3683. doi: 10.1016/j.bpj.2010.10.042, PMID: 21112292 PMC2998624

[ref145] Sempionatto J. R. Lin M. Yin L. De La Paz E. Pei K. Sonsa-Ard T.. (2021). An epidermal patch for the simultaneous monitoring of haemodynamic and metabolic biomarkers. Nat. Biomed. Eng. 5, 737–748. doi: 10.1038/s41551-021-00685-1 33589782

[ref146] Sercombe L. Veerati T. Moheimani F. Wu S. Y. Sood A. K. Hua S. (2015). Advances and challenges of liposome assisted drug delivery. Front. Pharmacol. 6:286. doi: 10.3389/fphar.2015.00286 26648870 PMC4664963

[ref147] Sharma S. Rabbani S. A. Agarwal T. Baboota S. Pottoo F. H. Kadian R. (2021). Nanotechnology driven approaches for the management of Parkinson's disease: current status and future perspectives. Curr. Drug Metab. 22, 287–298. doi: 10.2174/1389200221666201124123405, PMID: 33234098

[ref148] Sirsi S. Borden M. (2009). Microbubble compositions, properties and biomedical applications. Bubble Sci. Eng. Technol. 1, 3–17. doi: 10.1179/175889709X446507 20574549 PMC2889676

[ref149] Sun T. Zhang Y. Power C. Alexander P. M. Sutton J. T. Aryal M.. (2017). Closed-loop control of targeted ultrasound drug delivery across the blood-brain/tumor barriers in a rat glioma model. Proc. Natl. Acad. Sci. USA 114, E10281–e10290. doi: 10.1073/pnas.1713328114, PMID: 29133392 PMC5715774

[ref150] Sych Y. Chernysheva M. Sumanovski L. T. Helmchen F. (2019). High-density multi-fiber photometry for studying large-scale brain circuit dynamics. Nat. Methods 16, 553–560. doi: 10.1038/s41592-019-0400-4, PMID: 31086339

[ref151] Tasaki I. (1995). Mechanical and thermal changes in the Torpedo electric organ associated with its postsynaptic potentials. Biochem. Biophys. Res. Commun. 215, 654–658. doi: 10.1006/bbrc.1995.2514, PMID: 7488005

[ref152] Taylor G. J. Heberle F. A. Seinfeld J. S. Katsaras J. Collier C. P. Sarles S. A. (2017). Capacitive detection of low-enthalpy, higher-order phase transitions in synthetic and natural composition lipid membranes. Langmuir 33, 10016–10026. doi: 10.1021/acs.langmuir.7b02022, PMID: 28810118

[ref153] Treat L. H. Mcdannold N. Vykhodtseva N. Zhang Y. Tam K. Hynynen K. (2007). Targeted delivery of doxorubicin to the rat brain at therapeutic levels using Mri-guided focused ultrasound. Int. J. Cancer 121, 901–907. doi: 10.1002/ijc.22732 17437269

[ref154] Treat L. H. Mcdannold N. Zhang Y. Vykhodtseva N. Hynynen K. (2012). Improved anti-tumor effect of liposomal doxorubicin after targeted blood-brain barrier disruption by Mri-guided focused ultrasound in rat glioma. Ultrasound Med. Biol. 38, 1716–1725. doi: 10.1016/j.ultrasmedbio.2012.04.015, PMID: 22818878 PMC3438387

[ref155] Tufail Y. Matyushov A. Baldwin N. Tauchmann M. L. Georges J. Yoshihiro A.. (2010). Transcranial pulsed ultrasound stimulates intact brain circuits. Neuron 66, 681–694. doi: 10.1016/j.neuron.2010.05.008, PMID: 20547127

[ref156] Tyler W. J. (2012). The mechanobiology of brain function. Nat. Rev. Neurosci. 13, 867–878. doi: 10.1038/nrn3383 23165263

[ref157] Tyler W. J. Tufail Y. Finsterwald M. Tauchmann M. L. Olson E. J. Majestic C. (2008). Remote excitation of neuronal circuits using low-intensity, low-frequency ultrasound. PLoS One 3:e3511. doi: 10.1371/journal.pone.0003511, PMID: 18958151 PMC2568804

[ref158] Vigen K. K. Jarrard J. Rieke V. Frisoli J. Daniel B. L. Butts Pauly K. (2006). In vivo porcine liver radiofrequency ablation with simultaneous Mr temperature imaging. J. Magn. Reson. Imaging 23, 578–584. doi: 10.1002/jmri.20528, PMID: 16508928

[ref159] Wang C. Chen X. Wang L. Makihata M. Liu H. C. Zhou T.. (2022). Bioadhesive ultrasound for long-term continuous imaging of diverse organs. Science 377, 517–523. doi: 10.1126/science.abo2542, PMID: 35901155

[ref160] Wang H. Sun Y. Wang Y. Chen Y. Ge Y. Yuan J.. (2023). Temperature-controlled hyperthermia with non-invasive temperature monitoring through speed of sound imaging. Appl. Sci. 13:7317. doi: 10.3390/app13127317

[ref161] Wang D. Tai P. W. L. Gao G. (2019). Adeno-associated virus vector as a platform for gene therapy delivery. Nat. Rev. Drug Discov. 18, 358–378. doi: 10.1038/s41573-019-0012-9, PMID: 30710128 PMC6927556

[ref162] Watson D. S. Endsley A. N. Huang L. (2012). Design considerations for liposomal vaccines: influence of formulation parameters on antibody and cell-mediated immune responses to liposome associated antigens. Vaccine 30, 2256–2272. doi: 10.1016/j.vaccine.2012.01.070, PMID: 22306376 PMC3296885

[ref163] Wattiez N. Constans C. Deffieux T. Daye P. M. Tanter M. Aubry J. F.. (2017). Transcranial ultrasonic stimulation modulates single-neuron discharge in macaques performing an antisaccade task. Brain Stimul. 10, 1024–1031. doi: 10.1016/j.brs.2017.07.007, PMID: 28789857

[ref164] Wu D. Chen Q. Chen X. Han F. Chen Z. Wang Y. (2023). The blood-brain barrier: structure, regulation, and drug delivery. Signal Transduct. Target. Ther. 8:217. doi: 10.1038/s41392-023-01481-w, PMID: 37231000 PMC10212980

[ref165] Xie Y. Bagby T. R. Cohen M. S. Forrest M. L. (2009). Drug delivery to the lymphatic system: importance in future cancer diagnosis and therapies. Expert Opin. Drug Deliv. 6, 785–792. doi: 10.1517/17425240903085128, PMID: 19563270 PMC3102644

[ref166] Yang P. F. Phipps M. A. Jonathan S. Newton A. T. Byun N. Gore J. C.. (2021). Bidirectional and state-dependent modulation of brain activity by transcranial focused ultrasound in non-human primates. Brain Stimul. 14, 261–272. doi: 10.1016/j.brs.2021.01.006, PMID: 33460838 PMC7988301

[ref167] Yang H. Yuan Y. Wang X. Li X. (2020). Closed-loop transcranial ultrasound stimulation for real-time non-invasive Neuromodulation in vivo. Front. Neurosci. 14:445. doi: 10.3389/fnins.2020.00445, PMID: 32477055 PMC7235408

[ref168] Yoo S. S. Bystritsky A. Lee J. H. Zhang Y. Fischer K. Min B. K.. (2011). Focused ultrasound modulates region-specific brain activity. NeuroImage 56, 1267–1275. doi: 10.1016/j.neuroimage.2011.02.058, PMID: 21354315 PMC3342684

[ref169] Yuan Y. Wang Z. Liu M. Shoham S. (2020). Cortical hemodynamic responses induced by low-intensity transcranial ultrasound stimulation of mouse cortex. NeuroImage 211:116597. doi: 10.1016/j.neuroimage.2020.116597, PMID: 32018004

[ref170] Zaiss A. K. Cotter M. J. White L. R. Clark S. A. Wong N. C. Holers V. M.. (2008). Complement is an essential component of the immune response to adeno-associated virus vectors. J. Virol. 82, 2727–2740. doi: 10.1128/JVI.01990-07, PMID: 18199646 PMC2259003

[ref171] Zhang Y. Ren L. Liu K. Tong S. Yuan T. F. Sun J. (2021). Transcranial ultrasound stimulation of the human motor cortex. iScience 24:103429. doi: 10.1016/j.isci.2021.103429, PMID: 34901788 PMC8637484

[ref172] Zhao L. Feng Y. Hu H. Shi A. Zhang L. Wan M. (2016). Low-intensity pulsed ultrasound enhances nerve growth factor-induced neurite outgrowth through Mechanotransduction-mediated Erk1/2-Creb-Trx-1 signaling. Ultrasound Med. Biol. 42, 2914–2925. doi: 10.1016/j.ultrasmedbio.2016.07.017, PMID: 27592560

[ref173] Zhao Z. Nelson A. R. Betsholtz C. Zlokovic B. V. (2015). Establishment and dysfunction of the blood-brain barrier. Cell 163, 1064–1078. doi: 10.1016/j.cell.2015.10.067, PMID: 26590417 PMC4655822

[ref174] Zhong C. Guo N. Hu C. Ni R. Zhang X. Meng Z.. (2024). Efficacy of wearable low-intensity pulsed ultrasound treatment in the movement disorder in Parkinson's disease (the Swump trial): protocol for a single-site, double-blind, randomized controlled trial. Trials 25:275. doi: 10.1186/s13063-024-08092-y, PMID: 38650028 PMC11036625

[ref175] Zhou Y. F. (2011). High intensity focused ultrasound in clinical tumor ablation. World J. Clin. Oncol. 2, 8–27. doi: 10.5306/wjco.v2.i1.8, PMID: 21603311 PMC3095464

[ref176] Zhou S. Gao X. Park G. Yang X. Qi B. Lin M.. (2024). Transcranial volumetric imaging using a conformal ultrasound patch. Nature 629, 810–818. doi: 10.1038/s41586-024-07381-5 38778234 PMC11875229

[ref177] Zhou H. Meng L. Xia X. Lin Z. Zhou W. Pang N.. (2021). Transcranial ultrasound stimulation suppresses Neuroinflammation in a chronic mouse model of Parkinson's disease. IEEE Trans. Biomed. Eng. 68, 3375–3387. doi: 10.1109/TBME.2021.3071807 33830916

[ref178] Zhou H. Niu L. Xia X. Lin Z. Liu X. Su M.. (2019). Wearable ultrasound improves motor function in an Mptp mouse model of Parkinson's disease. IEEE Trans. Biomed. Eng. 66, 3006–3013. doi: 10.1109/TBME.2019.2899631, PMID: 30794160

